# Altered medial prefrontal cortex and dorsal raphé activity predict genotype and correlate with abnormal learning behavior in a mouse model of autism‐associated 2p16.3 deletion

**DOI:** 10.1002/aur.2685

**Published:** 2022-02-10

**Authors:** Rebecca B. Hughes, Jayde Whittingham‐Dowd, Steven J. Clapcote, Susan J. Broughton, Neil Dawson

**Affiliations:** ^1^ Division of Biomedical and Life Sciences, Faculty of Health and Medicine Lancaster University Lancaster UK; ^2^ School of Biomedical Sciences University of Leeds Leeds UK

**Keywords:** cognitive neuroscience, copy number variation/copy number variants, frontal lobe, genotype–phenotype correlation, imaging genetics, mouse models, serotonin

## Abstract

2p16.3 deletion, involving *NEUREXIN1* (*NRXN1*) heterozygous deletion, substantially increases the risk of developing autism and other neurodevelopmental disorders. We have a poor understanding of how *NRXN1* heterozygosity impacts on brain function and cognition to increase the risk of developing the disorder. Here we characterize the impact of *Nrxn1α* heterozygosity on cerebral metabolism, in mice, using ^14^C‐2‐deoxyglucose imaging. We also assess performance in an olfactory‐based discrimination and reversal learning (OB‐DaRL) task and locomotor activity. We use decision tree classifiers to test the predictive relationship between cerebral metabolism and *Nrxn1α* genotype. Our data show that *Nrxn1α* heterozygosity induces prefrontal cortex (medial prelimbic cortex, mPrL) hypometabolism and a contrasting dorsal raphé nucleus (DRN) hypermetabolism. Metabolism in these regions allows for the predictive classification of *Nrxn1α* genotype. Consistent with reduced mPrL glucose utilization, prefrontal cortex insulin receptor signaling is decreased in *Nrxn1α* ^*+/−*^ mice. Behaviorally, *Nrxn1α* ^+/−^ mice show enhanced learning of a novel discrimination, impaired reversal learning and an increased latency to make correct choices. In addition, male *Nrxn1α* ^+/−^ mice show hyperlocomotor activity. Correlative analysis suggests that mPrL hypometabolism contributes to the enhanced novel odor discrimination seen in *Nrxn1α* ^*+/−*^ mice, while DRN hypermetabolism contributes to their increased latency in making correct choices. The data show that *Nrxn1*α heterozygosity impacts on prefrontal cortex and serotonin system function, which contribute to the cognitive alterations seen in these animals. The data suggest that *Nrxn1α* ^*+/−*^ mice provide a translational model for the cognitive and behavioral alterations seen in autism and other neurodevelopmental disorders associated with 2p16.3 deletion.

**Lay Summary:**

Deletion of the chromosomal region 2p16.3, involving reduced *NEUREXIN1* gene expression, dramatically increases the risk of developing autism. Here, we show that reduced *Neurexin1α* expression, in mice, impacts on the prefrontal cortex and impairs cognitive flexibility. The data suggest that 2p16.3 deletion increases the risk of developing autism by impacting on the prefrontal cortex. Mice with the deletion are a useful model for testing new drugs to treat the cognitive flexibility problems experienced by people with autism.

## INTRODUCTION

Copy number variants (CNVs) are strongly implicated in the genetic etiology of autism and other neurodevelopmental disorders. Population‐based studies show that 2p16.3 deletions (OMIM #614332), involving heterozygous *NEUREXIN1* (*NRXN1*) gene deletion, are associated with developmental delay, learning difficulties, and a substantially increased risk of developing autism spectrum disorder (ASD, odds ratio = ~14.9, Matsunami et al., 2013; Wang et al., 2017; Yuen et al., 2017). Individuals with *NRXN1* deletions also show symptoms of attention deficit hyperactivity disorder (ADHD, Ching et al., 2010; Schaaf et al., 2012), obsessive compulsive disorder (OCD) (Castronovo et al., 2020) and are at increased risk of developing Tourette's syndrome (TS, odds ratio = ~20.3, Huang et al., 2017) and schizophrenia (ScZ, odds ratio = ~14.4, Marshall et al., 2017). Most *NRXN1* deletions localize to the promoter and initial exons of *NRXN1α*, leaving the *NRXN1β* coding sequence intact, and are thus predicted to impact on *NRXN1α* but not *NRXN1β* transcripts (Reichelt et al., 2012).

Rodent translational studies have been dedicated to elucidating the behavioral consequences of *Nrxn1α* deficiency, to establish if this results in phenotypes relevant to ASD and the other neurodevelopmental disorders associated with 2p16.3 deletion. Adult *Nrxn1α* homozygous knockout (KO) mice show decreased sociability, increased anxiety‐like behavior (Grayton et al., 2013) and a deficit in prepulse inhibition (PPI, Etherton et al., 2009), mirroring that seen in people with ASD (Cheng et al., 2018), ScZ (Braff et al., 1999), and TS (Swerdlow et al., 2001). Studies conducted across early development have identified deficits in ultrasonic vocalizations (USVs) and developmental milestones in *Nrxn1* KO mice, with some observations being sex specific (Armstrong et al., 2020). As *NRXN1* deletions in ASD, ScZ, and TS are commonly heterozygous, other studies have focused on characterizing *Nrxn1α* heterozygous (*Nrxn1α* ^*+/−*^) mice, reporting sex‐dependent alterations in novelty responsiveness (Laarakker et al., 2012) and memory deficits (Dachtler et al., 2015). *Nrxn1α* ^*+/−*^ mice also show deficits in social memory, while effects on sociability appear to be limited (Dachtler et al., 2015; Grayton et al., 2013). Emerging evidence suggests that the impact of *Nrxn1α* heterozygosity on cognition may be domain specific and may involve abnormally enhanced as well as impaired cognition, including enhanced motor learning (Hughes et al., 2020). Despite these observations in relatively simple tests, the potential impact of *Nrxn1α* heterozygosity on discrimination/associative learning and cognitive flexibility has not been assessed. This is surprising given the altered discrimination learning (Plaisted et al., 1998), associative learning (Brambilla et al., 2011; Eordegh et al., 2020) and cognitive flexibility, including impaired reversal learning (Crawley et al., 2020; Lange et al., 2017; Schlagenhauf et al., 2014), reported in neurodevelopmental disorders associated with 2p16.3 deletion.

Our previous study has identified alterations in functional brain network connectivity in *Nrxn1α* ^*+/−*^ mice that have translational relevance to those seen in neurodevelopmental disorders associated with 2p16.3 deletion (Hughes et al., 2020). How alterations in brain function relate to altered behavior in *Nrxn1α* ^*+/−*^ mice is yet to be characterized. In addition, we do not know how *Nrxn1α* heterozygosity in mice impacts on associative and reversal learning. Therefore, in this study, we characterize the performance of *Nrxn1α* ^*+/−*^ mice in an odor‐based discrimination and reversal learning (OB‐DaRL) task and characterize cerebral metabolism in the same animals using ^14^C‐2‐deoxyglucose (^14^C‐2‐DG) functional brain imaging, a translational analogue of ^18^F‐2‐deoxyglucose positron emission tomography (^18^F‐FDG PET) functional brain imaging routinely applied in humans. In addition, we assess the impact of *Nrxn1α* heterozygosity on locomotor and anxiety‐like behavior in the open‐field test (OFT).

## METHODS

### Animals

Full animal details can be found in Dachtler et al. (2015), and the generation of the mice is detailed in Missler et al. (2003). Male B6;129‐*Nrxn3* ^tm1Sud^/*Nrxn1* ^tm1Sud^/*Nrxn2* ^tm1Sud^/J mice (The Jackson Laboratory, Stock ID: #006377) were purchased as heterozygous KO at *Nrxn1*, homozygous KO at *Nrxn2*, and wild‐type (WT) at *Nrxn3* and backcrossed twice onto the C57BL/6NCrl strain (Charles River) to obtain mice selectively heterozygous for *Nrxn1α* (i.e., *heterozygous KO at Nrxn1α and WT at both Nrxn2* and *Nrxn3)*. Experimental animals (*Nrxn1α* ^*+/−*^ and WT littermates, aged 12–24 weeks) were generated through cousin mating. For *Nrxn1α* genotyping, the primers 5’‐CTG ATG GTA CAG GGC AGT AGA GGA CCA‐3′ (common forward), 5’‐CGA GCC TCC CAA CAG CGG TGG CGG GA‐3′ (WT reverse), and 5′‐GAG CGC GCG CGG CGG AGT TGT TGA C‐3′ (KO reverse) were used with HotShot Diamond (Clent Life Science) using a thermocycling program of: 95°C for 5 min, followed by 35 cycles of 94°C for 30 s, 70°C for 30 s, and 72°C for 30 s followed by a 72°C for 2 min step. PCR products were visualized using agarose gel electrophoresis, with a 440‐bp band indicating the wild‐type (WT) allele and a 390‐bp band indicating the KO allele. Animal genotype was determined from an identifying ear notch taken at weaning age and confirmed from a separate tissue sample taken at sacrifice. Animals were group housed (4–6/cage) under standard conditions (individually ventilated cages, 21°C, 45%–65% humidity, 12 h:12 h dark/light cycle, lights on at 07:00) with food and water access ad libitum. To motivate animals to perform in the OB‐DaRL task, where animals have to dig for a 14 mg sucrose reward pellet (TestDiet), mice were food restricted from 14 days prior to the start of the task until the end of the experiment. To allow the close monitoring of food consumption, animals were singly housed during restriction. Animals were given standard laboratory chow (Teklad maintenance diet, Envigo) and restricted to 80%–85% of their free‐feeding weight. Body weight and body condition score (Ullman‐Cullere & Foltz, 1999) were monitored 5 days a week. Behavioral experiments were conducted during the light phase. Experiments were carried out in compliance with the UK Animals (Scientific Procedures) Act, 1986 Amendment Regulations (SI 2012/3039), and approved by the Lancaster University Animal Welfare and Ethics Review Board (AWERB).

### Open field test

Locomotor activity (LMA) and anxiety‐like behaviors during exposure to a novel environment were recorded by placing the animals into white Perspex circular open field arenas (38 cm diameter) and video recording behavior for 15 min. Arenas were cleaned with 70% ethanol prior to and after each run. Automated tracking software (Ethovision XT v8.5, Noldus) was used to determine measures of LMA (distance moved (cm), average velocity (cm/s) and movement duration and frequency) and anxiety‐like behavior (central zone (15 cm diameter) duration (s) and frequency). Group sizes were WT: *n* = 36 (male, *n* = 18) and *Nrxn1α* ^*+/−*^: *n* = 33 (male, *n* = 15).

### OB‐DaRL task

A detailed overview of the OB‐DaRL task methodology is provided in the supplemental information, with the procedure adapted from published work in rats (Birrell & Brown, 2000; Dawson et al., 2012) and mice (Tanaka et al., 2011; Young et al., 2010). In brief, animals went through a series of task stages; *phase 1*: habituation and learning to dig, *phase 2*: odor habituation and novel odor discrimination, *phase 3*: a second odor discrimination and reversal learning. During *phase 1* animals learn to dig in sand in order to retrieve sucrose pellet rewards. In *phase 2*, animals were habituated to each odor (spices in sand, two odor bowls per trial, presented sequentially). Then animals undertake a novel odor discrimination (Discrimination 1), where they learn that only one of the two odors presented is rewarded. No reversal‐learning stage was applied following this initial discrimination. Instead, animals proceeded to *phase 3*, in which mice were presented with a new odor pair and learned the second odor‐reward discrimination (Discrimination 2). The animal then moved to the reversal‐learning stage, where the previously unrewarded odor within the odor pair used during Discrimination 2 was now rewarded, and vice versa. At each stage, the animal was judged to have learned the discrimination/reversal when it had performed six consecutive correct choices. This criterion is aligned with that applied in previous studies (Birrell & Brown, 2000; Dawson et al., 2012; Tanaka et al., 2011; Young et al., 2010). There is a 1.56% chance of animals performing six consecutive correct choice trials by random choice selection. At each phase, correct and incorrect trial choices, trials to criteria (TTC), and the latency to dig were recorded. In addition, the correction ratio, the proportion of trials that were correct choices made after a previously punished (exclusion from reward collection and a time out) incorrect trial was calculated. Group sizes were WT: *n* = 29 (male, *n* = 16) and *Nrxn1α* ^*+*/−^: *n* = 26 (male, *n* = 13).

### ^14^C‐2‐DG functional brain imaging

^14^C‐2‐DG functional brain imaging was conducted in accordance with published protocols (Hughes et al., 2020; Openshaw et al., 2020). The method used allows the determination of local cerebral glucose utilization (LCGU) in unanesthetised, awake, and freely‐moving mice. LCGU was determined in 39 brain regions of interest (RoI) across a range of neural systems (Table S2), from autoradiographic images using a computer‐based image analysis system (MCID/M5+, Interfocus) with reference to a stereotaxic mouse brain atlas (Paxinos & Franklin, 2001). The experimenter undertaking the analysis was blind to the sex and genotype of the animals. Group sizes were WT: *n* = 19 (male, *n* = 9) and *Nrxn1α* ^*+/−*^: *n* = 20 (male, *n* = 10). Further detail is provided in the supplementary information.

### Insulin receptor signaling characterization using Western blotting

Given the reduce glucose utilization we identified in the mPFC of *Nrxn1α* ^+/−^ mice, and the reduced PFC insulin signaling (Zhao et al., 2006) and insulin resistance (Manco et al., 2021) seen in disorders associated with 2p16.3 deletion, we used Western blotting to characterize insulin receptor signaling in the PFC and hippocampus of *Nrxn1α* ^+/−^ mice (WT: *n* = 6 [male, *n* = 3]; *Nrxn1α* ^+/−^: *n* = 8 [male, *n* = 4]). The primary antibodies were for the insulin receptor (beta subunit: IRβ, Merck Millipore, Cat. No. 07–724, 1:1000) and the phosphorylated/activated insulin receptor (tyrosine 972 phosphorylated, IR pTyr972/pIR, Merck Millipore, Cat. No. 07–838, 1:1000). The secondary antibody was a goat anti‐rabbit IgG, HRP‐linked Antibody (Cell Signaling, Cat. No. 7074S, 1:2000). Images were acquired with the Chemi‐Doc MP system and analyzed using Image Lab (Bio‐Rad Laboratories Ltd), with normalization to total protein load. Data were then normalized to same‐sex WT control. Full details are provided in the supplemental information.

### *Predicting* Nrxn1α *genotype from ^14^C‐2‐deoxyglucose functional brain imaging data using decision tree classification*

Decision tree classifiers, based on the classification and regression trees (CART) algorithm, are a supervised machine learning approach that builds a classifier from a set of training samples with a list of known features and known class labels. In terms of our data, the class label was genotype (WT or *Nrxn1α* ^*+/−*^) and the feature measurements were LCGU in 39 RoI. The decision trees can then be used to predict the classification of unknown “test” cases. The trees also provide information on which features are most useful in the classification and provide threshold values that the algorithm uses to perform the classification. To determine the parameters that resulted in optimal tree performance, we characterized the ability of the trees to classify unseen data in terms of accuracy (the ability to correctly classify animals), sensitivity (the ability to correctly classify true positives, i.e. *Nrxn1α* ^*+/−*^ mice), and specificity (the ability to correctly classify true negatives, i.e. WT mice) using a leave‐one‐out cross validation (LOOCV) strategy. We then tested the ability of these trees to correctly classify mice from an independently generated test set involving 8 WT (male *n* = 4) and 6 *Nrxn1α* ^*+/−*^ (male *n* = 3) mice. Performance was assessed in terms of accuracy, precision (the ability of the classifier not to mislabel a case), and specificity. Scikit‐learn (https://sklearn.org, Pedregosa et al., 2011) was used to undertake decision tree classification. Further detail is included in the supplemental information.

### Statistical analysis

Data were statistically analyzed in R (https://www.R-project.org/). OFT data were analyzed using repeated measures ANOVA with sex and genotype as independent variables and time bin as the within subjects variable. Where significant sex *x* genotype interactions were observed, data from each sex were analyzed separately using repeated measures ANOVA. OB‐DaRL task and ^14^C‐2‐DG data were analyzed using ANOVA, with sex and genotype as independent variables. For genotype effects in the ^14^C‐2‐DG data, we report both the unadjusted p‐value from the ANOVA and the p‐value adjusted using post hoc Benjamini‐Hochberg correction for multiple comparisons on the basis of comparing 39 brain RoI. Western blot data were analyzed using Wilcoxon test. The relationship between LCGU and behavioral parameters was analyzed using Pearson's correlation coefficient. Significance was set at p < 0.05.

## RESULTS

### Nrxn1α *heterozygosity increases anxiety‐like behavior in mice of both sexes and induces a mild hyperactivity selectively in male mice*

**FIGURE 1:**
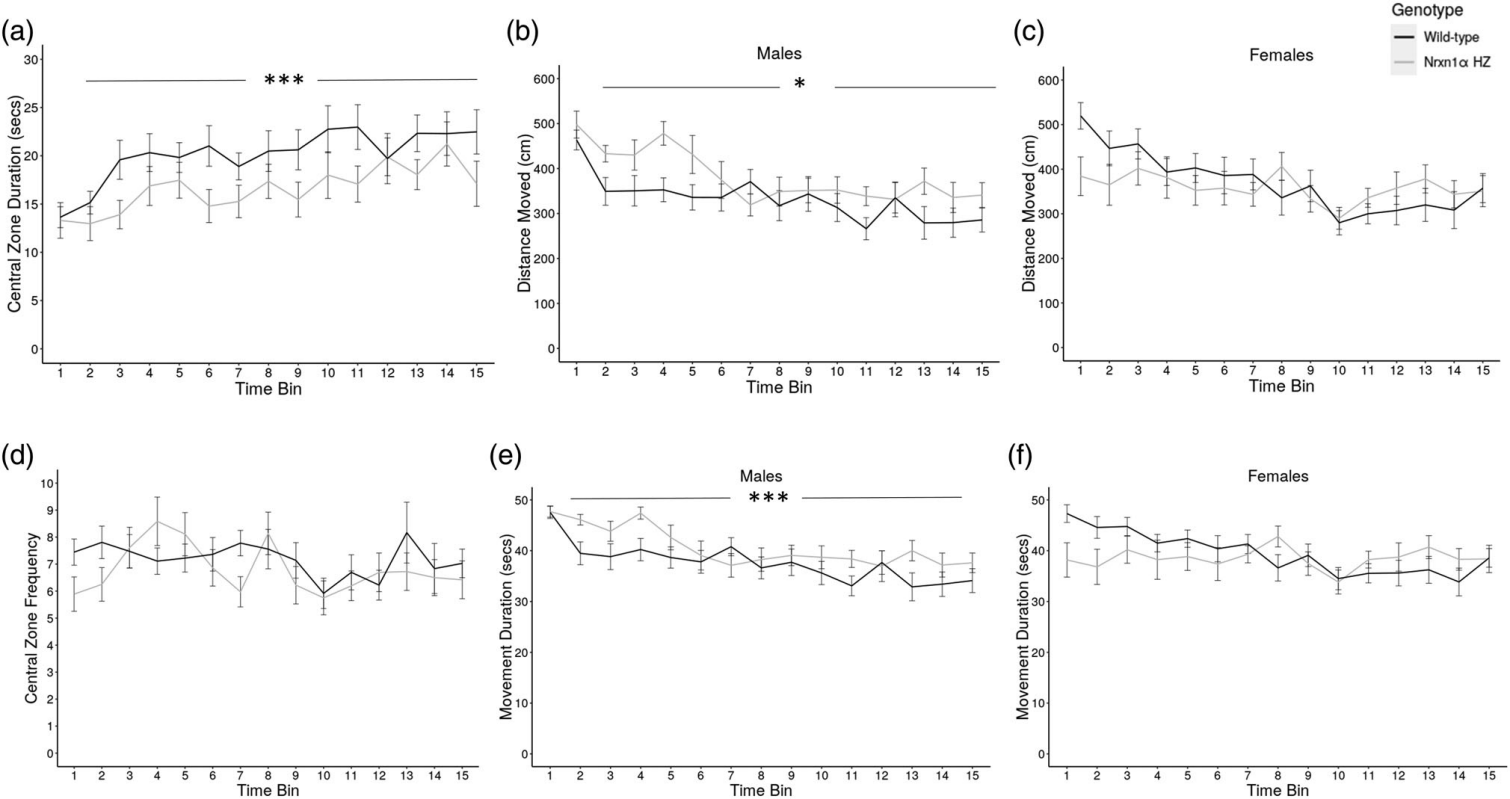
*Nrxn1α* ^*+/−*^ mice show altered anxiety‐like behavior and locomotor activity in the open‐field test (OFT). *Nrxn1α* ^*+/−*^ mice show (a) reduced time spent in the central zone (s) but not an altered (d) frequency of movement into the central zone of the open field arena. Male, but not female, *Nrxn1α* ^*+/−*^ mice show hyperactivity in the open field as evidenced by increased (b, c) distance moved (cm) and (e, f) movement duration (s). Data shown as mean ± SE over the 15 min recording duration (60 s time bins). *p < 0.05 and ***p < 0.001 genotype effect (repeated measures ANOVA). Group sizes were WT: *N* = 36 (male, *n* = 18) and *Nrxn1α* ^*+/−*^: *N* = 33 (male, *n* = 15). WT = wild‐type and *Nrxn1α* Hz = *Nrxn1α* ^*+/−*^ mice

When exposed to a novel open field environment, *Nrxn1α* ^*+/−*^ mice showed increased anxiety‐like behavior, evidenced by less time spent in the arena central zone (Figure 1, F_(1960)_ = 16.72, p < 0.001). By contrast, the frequency with which the animals entered the central zone was not different between genotypes (F_(1974)_ = 0.19, p = 0.664). There was no evidence that sex influenced the impact on *Nrxn1α* heterozygosity on time spent in the central zone (F_(1960)_ = 0.01, p = 0.906). In addition, male *Nrxn1α* ^*+/−*^ mice showed a mild hyperactivity when exposed to the novel open field environment, while female mice did not. A significant sex x genotype interaction was found for distanced traveled (F_(1974)_ = 12.50, p < 0.001). Male *Nrxn1α* ^*+/−*^ mice showed an increased distanced traveled (15% increase, F_(1464)_ = 14.99, p < 0.001) in comparison to male WTs. By contrast, female *Nrxn1α* ^*+/−*^ mice showed a similar distanced traveled (F_(1509)_ = 1.00, p = 0.317) to female WT controls. While the hyperlocomotor activity seen in male *Nrxn1α* ^*+/−*^ mice appeared to be more evident during early time bins of the OFT, we found no evidence for a significant interaction between time bin and genotype for distance traveled (F_(14,464)_ = 1.07, p = 0.381). Therefore, there was no evidence that the increased activity was limited to specific time point during the OFT. A similar effect was seen for movement duration (sex *x* genotype, F_(1974)_ = 11.22, p < 0.001), with *Nrxn1α* ^*+/−*^ males (8% increase, F_(1464)_ = 11.16, p < 0.001) but not females (F_(1509)_ = 1.42, p = 0.234) being significantly different from same‐sex WTs. Again, in males, there was no significant interaction between genotype and time bin for movement duration (F_(14,464)_ = 0.99, p = 0.464), indicating that the increased activity was not limited a specific time point during the OFT. By contrast, there was no evidence that *Nrxn1*α heterozygosity impacted on movement frequency, in mice of either sex (Figure S1). These data show that *Nrxn1α* heterozygosity induces a mild hyperactivity selectively in males, in part by impacting on movement duration while not modifying the frequency at which movements are initiated.

### Nrxn1α *heterozygosity enhances learning of a novel odor‐based discrimination but impairs reversal learning*

**FIGURE 2:**
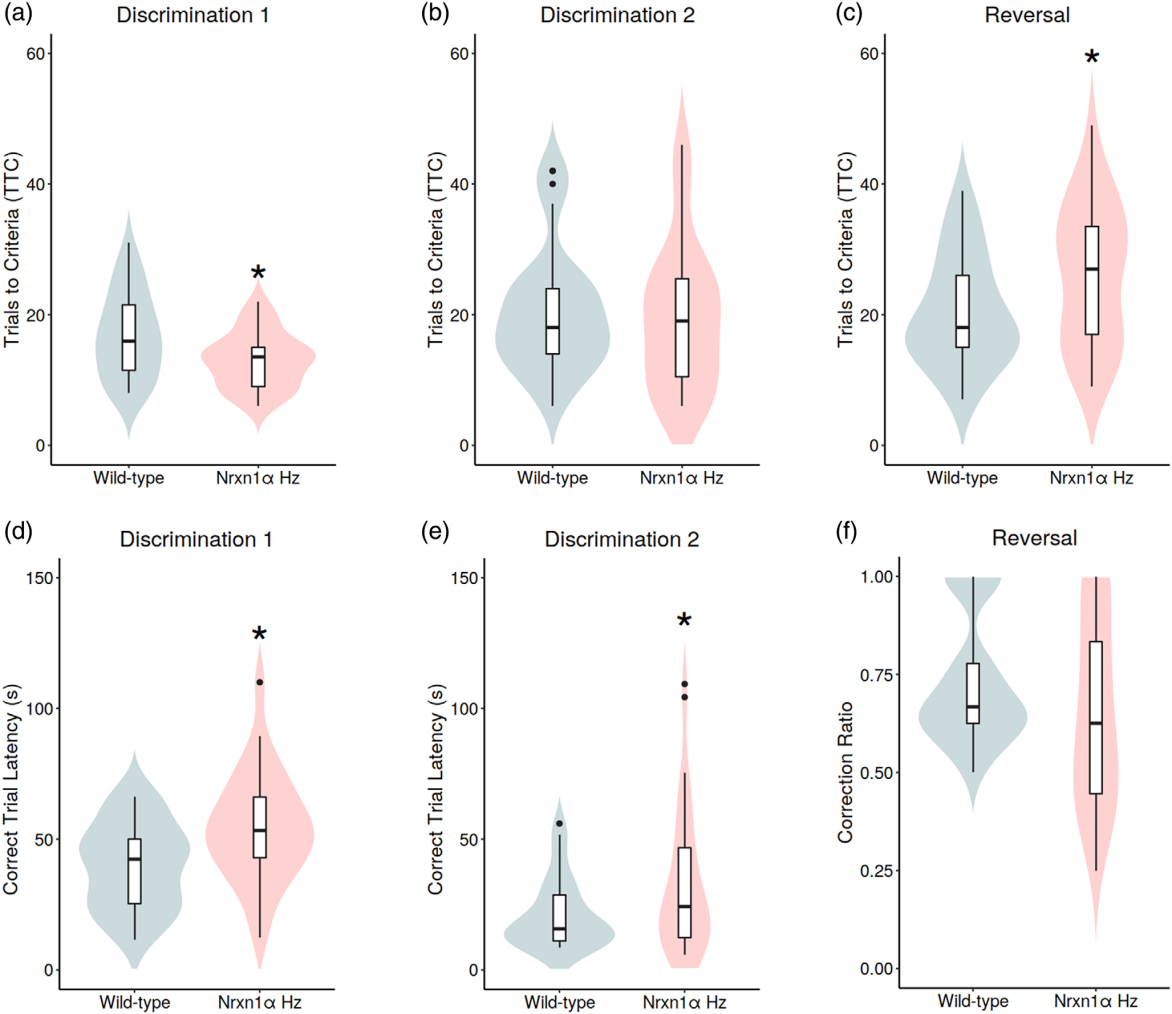
*Nrxn1α* ^*+/−*^ mice show enhanced learning of a novel odor‐reward discrimination but impaired reversal learning. *Nrxn1α* ^*+/−*^ mice show a reduced number of trials to reach criteria (TTC) during the (a) initial (Discrimination 1) but not (b) second (Discrimination 2) odor‐reward discrimination phase. (c) *Nrxn1α* ^*+/−*^ mice require an increased number of TTC during the reversal‐learning stage. *Nrxn1α* ^*+/−*^ mice show an increased latency to make correct, but not incorrect, responses during the (d) initial (Discrimination 1) and (e) second discrimination (Discrimination 2). *Nrxn1α* ^*+/−*^ mice do not show an altered sensitivity to punishment for making incorrect choices during the reversal phase, as shown by the (f) correction ratio. Data shown as violin plots. *p < 0.05 difference from WT (ANOVA). Group sizes were WT: *N* = 29 (male, *n* = 16) and *Nrxn1α* ^*+/−*^: *N* = 26 (male, *n* = 13). WT = wild‐type and *Nrxn1α* Hz = *Nrxn1α* ^*+/−*^ mice

In the OB‐DaRL task *Nrxn1α* ^*+/−*^ mice required fewer trials to reach criterion (TTC) during the first odor discrimination (Figure 2, Discrimination 1: F_(1,47)_ = 5.74, p = 0.021), indicating enhanced learning of this novel odor‐reward association. However, when presented with the second odor pair, *Nrxn1α* ^*+/−*^ mice performed at a similar level to WTs (Discrimination 2: TTC, F_(1,56)_ = 0.16, p = 0.694). There was no evidence that the impact of *Nrxn1α* heterozygosity was influenced by sex for either discrimination (sex *x* genotype; Discrimination 1: F_(1,47)_ = 0.18, p = 0.673; Discrimination 2: F_(1,56)_ = 0.57, p = 0.454).

During the initial discrimination *Nrxn1α* ^*+/−*^ mice took significantly longer to complete trials than WTs when making correct (trial latency; Discrimination 1: F_(1,48)_ = 7.69, p = 0.008) but not incorrect (F_(1,48)_ = 1.94, p = 0.170) choices. This genotype effect was also evident in the second odor discrimination (Discrimination 2, correct: F_(1,53)_ = 4.60, p = 0.037, incorrect: F_(1,49)_ = 2.04, p = 0.160). There was no evidence that the genotype difference in correct choice response latency was influenced by sex (Discrimination 1: F_(1,48)_ = 0.20, p = 0.657; Discrimination 2, F_(1,53)_ = 0.36, p = 0.550). The mild hyperactive phenotype identified in male *Nrxn1α* ^*+/−*^ mice could potentially contribute to the increased correct response latency seen, although this is unlikely to be the case for the increased latency seen in female *Nrxn1α* ^*+/−*^ mice, as they do not show this hyperactivity. We found no correlation between LMA levels in the open field, as measured by distance traveled, and correct trial latency during Discrimination 1 (r = 0.10, p = 0.491) or Discrimination 2 (r = −0.11, p = 0.481), suggesting that correct trial latency is not strongly related to LMA levels. This suggests that the mild hyperactivity phenotype seen in male *Nrxn1α* ^*+/−*^ mice is unlikely to be driving the increased correct trial latency seen in these animals.

In the reversal learning phase, *Nrxn1α* ^*+/−*^ mice required significantly more TTC (F_(1,47)_ = 4.21, p = 0.046) than WT mice, supporting a deficit in reversal learning. There was no evidence that this deficit was influenced by sex (TTC, F_(1,47)_ = 1.46, p = 0.233). To determine whether this deficit was due to an altered sensitivity to punishment, we characterized the correction ratio (Figure 2). We found no evidence to support a difference in the correction ratio between *Nrxn1α* ^*+/−*^ and WT mice in the reversal phase (F_(1,45)_ = 1.88, p = 0.177). This was also true for the first (F_(1,38)_ = 0.15, p = 0.704) and second (F_(1,45)_ = 2.03, p = 0.161) odor discrimination (Figure S2). These data support a similar sensitivity to punishment in *Nrxn1α* ^*+/−*^ mice.

We found no evidence to suggest that performance during the reversal learning stage of the task, in terms of TTC, was significantly correlated with performance during learning the odor discrimination in Discrimination 2. In this way, TTC in the reversal stage did not correlate with TTC (r = 0.19, p = 0.243) or trial response latencies (correct [r = 0.22, p = 0.166] or incorrect [r = 0.07, p = 0.660]) in Discrimination 2. This suggests that any increase in odor exposure during Discrimination 2 that results from the increased correct trial latency seen in *Nrxn1α* ^*+/−*^ mice is unlikely to contribute to their altered performance in the reversal learning stage.

### *Cerebral metabolism in the prefrontal‐mesolimbic system and dorsal raphé predicts* Nrxn1α *heterozygosity*

**FIGURE 3:**
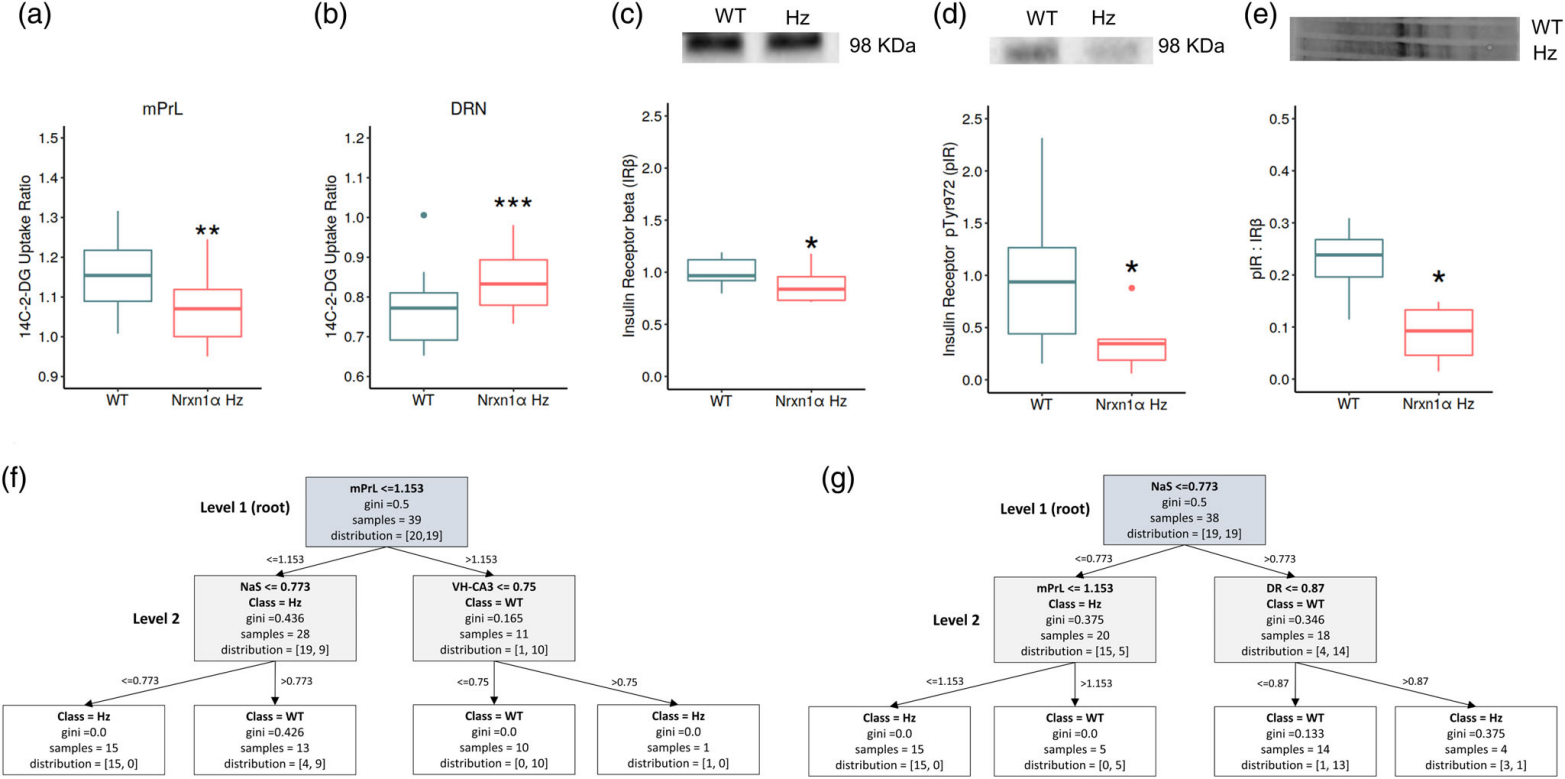
Metabolism in the mPrL, DRN, and NaS predicts *Nrxn1α* genotype classification and insulin receptor signaling is decreased in the PFC of *Nrxn1α* ^*+/−*^ mice. *Nrxn1α* ^*+/−*^ mice show significant (a) hypometabolism in the medial prelimbic cortex (mPrL) with a contrasting (b) hypermetabolism in the dorsal raphé (DRN). **p < 0.05, ***p < 0.01 significant difference from WT (ANOVA). (c) Insulin receptor (IRβ) protein levels, (d) activated insulin receptor levels (pIR), and (e) the ratio between pIR:IRβ are significantly decreased in the PFC of *Nrxn1α* ^*+/−*^ mice. *p < 0.05 difference from WT (Wilcoxon). Inset blot images show (c) representative IRβ bands, (d) representative pIR bands, and (e) total protein levels for the same lanes. CART decision tree classification predicts *Nrxn1α* genotype on the basis of metabolism in the mPrL, DRN, and NaS. The two most common decision tree structures generated during LOOCV are shown, with the decision tree structure in (f) being present in 21% of the models generated and (g) being present in 18%. Full details of the other LOOCV models generated are shown in the supplemental information. Group sizes were WT: *N* = 19 (male, *n* = 9) and *Nrxn1α* ^*+/−*^: *N* = 20 (male, *n* = 10). WT = wild‐type and *Nrxn1α* Hz = *Nrxn1α* ^*+/−*^ mice

*Nrxn1α* ^*+/−*^ mice showed hypometabolism in the medial prelimbic cortex (mPrL: F_(1,31)_ = 10.73, p = 0.003 unadjusted, p = 0.049 Benjamini‐Hochberg corrected, 95% CI [−0.14, −0.03]) with a contrasting hypermetabolism in the dorsal raphé nucleus (DRN: F_(1,31)_ = 14.56, p < 0.001 unadjusted, p = 0.024 Benjamini‐Hochberg corrected, 95% CI [0.034, 0.1549], Figure 3). There was no evidence that sex modified the impact of *Nrxn1α* heterozgosity on cerebral metabolism in these regions (sex *x* genotype; mPrL: F_(1,31)_ = 0.47, p = 0.500; DRN: F_(1,31)_ = 0.04, p = 0.84). Full ^14^C‐2‐DG data are shown in the supplemental information (Table S2).

We used decision tree classification to establish the validity of regional cerebral metabolism in predicting *Nrxn1α* genotype. Initial validation studies, employing LOOCV, identified decision trees with a depth of 2, using the Gini impurity index (a measure of sample impurity at each node), as the most useful in classifying *Nrxn1α* genotype using the brain imaging data. These decision trees had an accuracy of 79%, a specificity of 68%, and a sensitivity of 90% in classifying *Nrxn1α* ^*+/−*^ from WT mice, when classifying the excluded case from LOOCV.

The decision trees most commonly seen in the LOOCV analysis (56%) featured the mPrL at the root (level 1) and the nucleus accumbens shell (NaS) at level two. The next most common decision trees involved NaS at the root (level 1) and the mPrL at level 2 (18%, Figure 3). The finding that mPrL metabolism can be used to classify *Nrxn1α* genotype is consistent with the mPrL hypometabolism seen in *Nrxn1α* ^*+/−*^ mice. However, there was no evidence for a significant difference in NaS metabolism between the genotypes, at the group level (F_(1,31)_ = 2.82, p = 0.103). As the NaS most commonly featured at level 2 of the decision trees, this suggests that metabolism in this region is useful in differentiating a subset of *Nrxn1α* ^*+/−*^ from WT mice, for example once mPFC metabolism has been taken into account. This may suggest that the NaS is dysfunctional in a subset of *Nrxn1α* ^*+/−*^ mice. To test this, we selectively analyzed genotype significance in a subset of animals when mPFC metabolism had been taken into account. As the decision trees indicated that a mPFC ^14^C‐2‐DG uptake ratio > 1.153 is associated with being WT (Figure 3), we analyzed group‐level genotype differences in NaS metabolism in mice with a mPFC ^14^C‐2‐DG uptake ratio < =1.153. We found evidence for NaS hypometabolism in this subgroup of *Nrxn1α* ^*+/−*^ mice (F_(1,20)_ = 5.62, p = 0.028, WT: *n* = 9, *Nrxn1α* ^*+/−*^: *n* = 19). This is consistent with the observation that a NaS ^14^C‐2‐DG uptake ratio < 0.773 is useful in classifying *Nrxn1α* ^*+/−*^ from WT mice, once mPrL metabolism has been considered (Figure 3). The next most common structure (13%) seen in the LOOCV decision trees involved the DRN at the root (level 1), with a DRN ^14^C‐2‐DG uptake ratio < =0.728 being indicative of the WT genotype. This is consistent with the significant DRN hypermetabolism found in *Nrxn1α* ^*+/−*^ mice.

To further validate these decision trees, we tested their ability to classify mice from an independently generated test set. For this test data, decision trees containing the mPrL (level 1) and NaS (level 2) performed well in classify *Nrxn1α* ^*+/−*^ from WT mice (accuracy: 79%–93%, precision: 71%–83%, specificity: 83%–100%). Decision trees involving the DRN at level 1 also performed well (accuracy: 71%–100%, precision: 83%, specificity: 62%). See the supplemental information for a detailed overview.

These data show that the mPrL and DRN are dysfunctional in *Nrxn1α* ^*+/−*^ mice, and that metabolism in these regions, along with that in the NaS, are useful in predicting *Nrxn1α* genotype.

### *Insulin receptor signaling is reduced in the PFC, but not in the hippocampus, of* ***Nrxn1α*** *^***+/−***^ mice*

We found evidence supporting insulin resistance in the PFC of *Nrxn1α* ^*+/−*^ mice, consistent with the decreased mPrL glucose utilization present in these animals (Figure 3, Table S2). Both total IR (IRβ, p = 0.033) and phosphorylated/activated IR (pIR: p = 0.047) levels were reduced in the PFC of *Nrxn1α* ^+/−^ mice. The ratio between pIR and IRβ was also reduced (p = 0.019). This supports both reduced IR expression and reduced IR activation in the PFC of *Nrxn1α* ^+/−^ mice. By contrast, IRβ and pIR levels, and the pIR:IRβ ratio, were not altered in the hippocampus of *Nrxn1α* ^+/−^ mice, consistent with the unaltered hippocampal glucose utilization seen in these animals (Table S2 and Figure S3).

### Constitutive cerebral metabolism in the mPFC and dorsal raphé correlates with measures of LMA and odor‐reward discrimination learning in mice

To further understand the relationship between the altered cerebral metabolism and behavior seen in *Nrxn1α* ^*+/−*^ mice, we undertook correlative analysis. Given the altered metabolism seen in the mPrL and DRN, we analyzed correlations between metabolism in these regions and the behavioral parameters found to be altered in *Nrxn1α* ^*+/−*^ mice.

**FIGURE 4:**
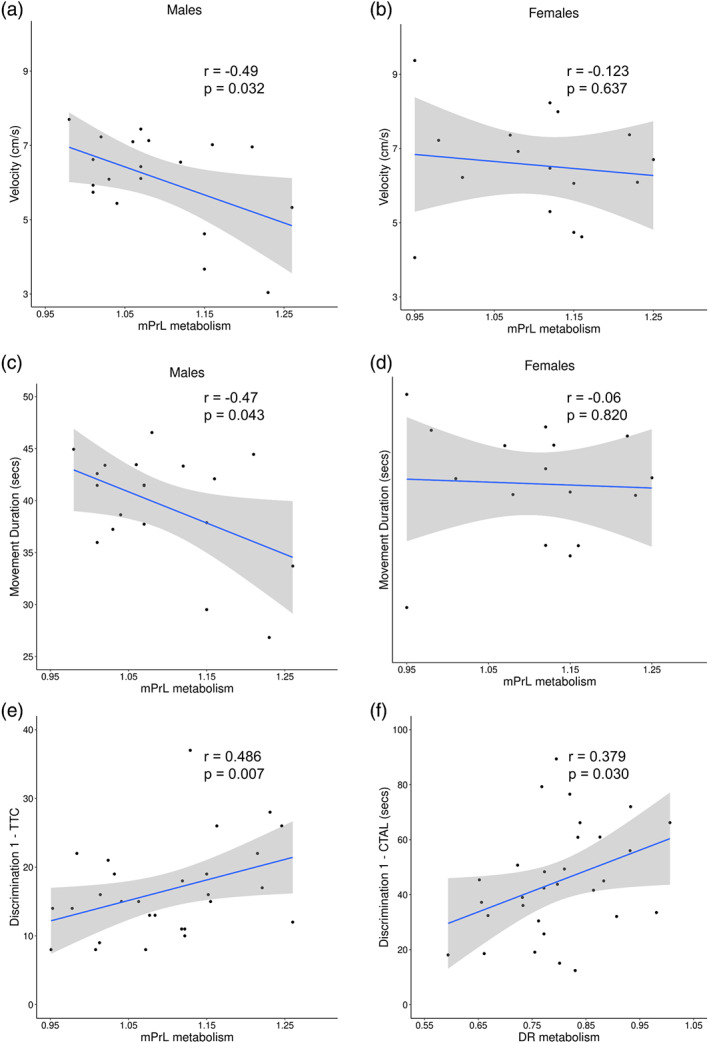
Metabolism in the mPrL and DRN correlate with behaviors altered in *Nrxn1α* ^*+/−*^ mice. Cerebral metabolism in the mPrL negatively correlates with locomotor activity measures, including (a, b) velocity and (c, d) movement duration (s) in male, but not in female, mice. (e) Cerebral metabolism in the mPrL positively correlates with trials to criteria (TTC) in the initial discrimination (Discrimination 1) phase of the OB‐DaRL task, in mice of both sexes. (f) Cerebral metabolism in the DRN positively correlates with correct choice trial latency in the initial discrimination (Discrimination 1) phase of the task, in mice of both sexes. Full data are shown in the supplemental information (Tables S3 and S4). Group sizes were WT: *N* = 19 (male, *n* = 9) and *Nrxn1α* ^*+/−*^: *N* = 20 (male, *n* = 10). WT = wild‐type and *Nrxn1α* Hz = *Nrxn1α* ^*+/−*^ mice

Cerebral metabolism in the mPrL negatively correlated with measures of LMA (distance moved: r = −0.49, p = 0.033; velocity: r = −0.49, p = 0.033, movement duration: r = 0.47, p = 0.04) in male mice. These correlations were not seen in female mice (distance moved: r = −0.13, p = 0.62; velocity: r = −0.12, p = 0.647; movement duration: r = −0.06, p = 0.820, Figure 4). There was no evidence that DRN metabolism correlates with LMA (Tables S3 and S4). This suggests that mPrL metabolism negatively correlates with LMA in male but not female mice. Thus the mPrL hypometabolism seen in *Nrxn1α* ^*+/−*^ mice may contribute to the hyperlocomotor activity that is selectively seen in male *Nrxn1α* ^*+/−*^ mice.

Metabolism in the mPrL positively correlated with the number of trials required to learn a novel odor discrimination (Discrimination 1, TTC, r = 0.48, p = 0.007). This suggests that the mPrL hypometabolism seen in *Nrxn1α* ^*+/−*^ mice may contribute to their enhanced performance in this phase of the task. By contrast, DRN metabolism did not correlate with TTC during this phase (r = −0.22, p = 0.255). However, DRN metabolism did positively correlate with response latency for making correct choices during this phase (r = 0.38, p = 0.030). Therefore, the DRN hypermetabolism seen in *Nrxn1α* ^*+/−*^ mice may relate to the longer latency to make correct choices seen in these animals. By contrast, mPrL metabolism did not correlate with correct choice latency during this phase of the task (r = −0.09, p = 0.602).

For the reversal phase of the task, there was no correlation between metabolism in the mPrL (r = −0.24, p = 0.222) or DRN (r = −0.04, p = 0.835) with TTC. This suggests that the altered metabolism seen in these regions may not relate to the reversal learning deficits seen in *Nrxn1α* ^*+/−*^ mice. Full data are shown in the supplemental information (Tables S3 and S4).

## DISCUSSION

Our data show that *Nrxn1α* heterozygosity results in a deficit in reversal learning and abnormally enhanced learning of a novel odor‐based discrimination that may have relevance to the core cognitive dysfunction seen in ASD and other neurodevelopmental disorders associated with 2p16.3 deletion. In addition, we show a sex‐specific impact of *Nrxn1α* heterozygosity on LMA, with effects being more pronounced in males. This is consistent with published data reporting sex differences in the behavioral impact of *Nrxn1α* deletion (Armstrong et al., 2020; Dachtler et al., 2015; Grayton et al., 2013; Laarakker et al., 2012) and the more pronounced symptomatology of disorders associated with 2p16.3 deletion, such as ASD, in males. We also show that *Nrxn1α* heterozygosity impacts on mPFC and DRN function, with metabolism in these regions being predictive of *Nrxn1α* genotype. In addition, metabolism in these regions correlates with the behavioral differences seen in *Nrxn1α* ^+/−^ mice. Overall, these data suggest that *Nrxn1α* ^+/−^ mice provide a useful translational model for further understanding the mechanisms through with 2p16.3 deletion impacts on the brain to increase the risk of ASD and other neurodevelopmental disorders. Moreover, this animal model provides a useful tool for validating novel treatments for the cognitive and behavioral symptoms experienced by individuals with 2p16.3 deletion, and neurodevelopmental disorders associated with the CNV.

### *The behavioral deficits seen in* Nrxn1α*^+/−^ mice have translational relevance to those seen in neurodevelopmental disorders associated with 2p16.3 deletion*

*Nrxn1α* heterozygosity in mice induces phenotypes that have translational relevance to those seen in individuals with 2p16.3 deletions and disorders associated with this CNV, including ASD. The abnormally enhanced learning of a novel odor‐reward discrimination in *Nrxn1α* ^*+/−*^ mice may have relevance to the enhanced abilities for stimulus discrimination (O'Riordan & Passetti, 2006) and for the discrimination of novel stimuli (Edmondson et al., 2020; Plaisted et al., 1998), reported in individuals with ASD. However, it should be noted that these have primarily been characterized in terms of sensitivity to visual and auditory stimuli in ASD, whereas here were reporting the effect on olfactory discrimination. Determining if visual or auditory discrimination is also enhanced in *Nrxn1α* ^*+/−*^ mice, for example though use of the visual discrimination and reversal learning touchscreen task (Bussey et al., 2012), would be of interest. Nevertheless, the observation of an enhanced novel odor‐based discrimination in *Nrxn1α* ^+/−^ mice is consistent with reports of enhanced learning, in terms of motor learning, as a consequence of *Nrxn1α* heterozygosity (Hughes et al., 2020) and knockout (Etherton et al., 2009). By contrast, there are also reports of impaired learning, in social (Dachtler et al., 2015) and novel object (Laarakker et al., 2012) recognition, in *Nrxn1α* mutant mice. This suggests that the altered learning induced by *Nrxn1α* heterozygosity is domain specific, with both enhanced and impaired learning evident based on the type of learning and memory being measured. Further characterization is needed to fully understand the basis of altered cognition induced by *Nrxn1α* heterozygosity and its translational alignment to 2p16.3 deletion in humans. The observation that *Nrxn1α* ^*+/−*^ mice require a similar number of trials to learn the second odor discrimination to WT mice suggests that their enhanced performance during the first odor discrimination relates to an enhanced ability to learn the contextual basis of the novel discrimination, rather than an enhanced ability to learn to discriminate between odor stimuli per se. As our study is the first reporting enhanced discrimination of a novel odor discrimination and a reversal learning deficit in *Nrxn1α* heterozygous mice, future studies confirming these cognitive deficits in other laboratories would be of interest.

In contrast to the enhanced novel discrimination learning, reversal learning was impaired in *Nrxn1α* ^+/−^ mice. This has translational relevance to the reversal learning deficit seen in neurodevelopmental disorders associated with 2p16.3 deletion, including that in ASD (Crawley et al., 2020), ScZ (Reddy et al., 2016; Schlagenhauf et al., 2014), and TS (Shephard et al., 2016). However, while these studies generally indicate perseveration and/or a lack of sensitivity to feedback as key mechanisms in the reversal learning deficit seen in these disorders (Crawley et al., 2020; Reddy et al., 2016), we found that sensitivity to punishment/feedback was not impaired in *Nrxn1α* ^+/−^ mice. Thus, alternative mechanisms may underlie the reversal learning deficit seen in *Nrxn1α* ^*+/−*^ mice. Given that set‐shifting deficits are also seen in disorders associated with 2p16.3 deletion (Jazbec et al., 2007; Westwood et al., 2016), future studies aimed at characterizing this potential deficit in *Nrxn1α* ^+/−^ mice would also be of translational interest.

Consistent with previous data (Laarakker et al., 2012), we identified hyperlocomotor activity when exposed to a novel environment selectively in male *Nrxn1α* ^+/−^ mice. However, these effects are not always found (Dachtler et al., 2015), and a contrasting hypoactivity has been reported in *Nrxn1α* KO mice (Grayton et al., 2013). This suggests that gene‐dosage effects are important, at least for some phenotypes. Therefore, *Nrxn1α* ^*+/−*^ mice may arguably represent a more translationally‐relevant model of the 2p16.3 deletions most commonly seen in humans than *Nrxn1α* KO mice, as these are heterozygous in nature. The hyperactive exploratory behavior seen in male *Nrxn1α* ^*+*/−^ mice may have translational relevance to the hyperactive exploratory behavior reported in disorders association with 2p16.3 deletion, including ADHD (Garcia Murillo et al., 2015) and ScZ (Perry et al., 2009). The observation that this mild hyperactivity is seen only in males may also have relevance to the increased prevalence of ASD in males. This, along with other recent data on the greater impact of 16p11.2 hemideletion on sleep, learning and brain structure in male as compared to female mice (Angelos et al., 2017; Grissom et al., 2018; Kumar et al., 2018), supports the suggestion that males may be more sensitive to ASD risk genes and chromosomal variations than females, at least in relation to some disease‐relevant phenotypes.

We also identified increased anxiety‐like behavior in *Nrxn1α* ^*+/−*^ mice, as evidenced by a decreased duration spent in the central zone of the arena. One concern is that this could be influenced by the mild hyperlocomotor activity seen selectively in male *Nrxn1α* ^*+/−*^ mice. However, as we found no significant difference in the frequency of entries into the central zone in *Nrxn1α* ^*+/−*^ mice, this suggests that increased LMA is not the primary driver of this observation. This is further supported by the observation that female *Nrxn1α* ^*+/−*^ mice do not show this hyperlocomotor activity, but do spend less time in the central zone of the open field arena. The interpretation that this observation is related to increased anxiety also needs to be made with some caution, particularly in the context of the available published data on the impact of *Nrxn1α* on anxiety‐like behaviors. The published data are mixed, with increased anxiety‐like behavior reported in *Nrxn1α* ^*−/−*^ (KO) but not *Nrxn1α* ^*+/−*^ mice in the OFT, elevated plus maze (EPM) and light–dark box (Grayton et al., 2013). By contrast, Etherton et al., (1999) found no significant anxiety‐like phenotype in the OFT and EPM in *Nrxn1α* ^*−/−*^ (KO) mice, and Dachtler et al., (2015) also found no significant anxiety‐like phenotype in *Nrxn1α* ^*+/−*^ mice in these tests. It is likely that differences in animal handling and the experimental protocols used contribute to these differential observations. In addition, differences in sample size are also likely to be relevant, with our study having the largest sample size to date. This suggests that, while our data support a potential increased anxiety‐like phenotype in *Nrxn1α* ^*+/−*^ mice, further experimental data are need to more firmly prove this phenotype.

### *mPFC and dorsal raphé metabolism correlate with behavioral alterations present in* Nrxn1a^+/−^ *mice and predict* Nrxn1α *genotype*

Our data indicate that the mPFC is hypoactive and the DRN hyperactive in *Nrxn1α* ^+/−^ mice, and that metabolism in these regions, along with that in other regions including the NaS, is useful in predicting *Nrxn1α* genotype. Hypofrontality, decreased PFC function, is seen in multiple neurodevelopmental disorders associated with 2p16.3 deletion and contributes to executive dysfunction (Hart et al., 2013; Hill et al., 2004; Mitelman et al., 2018; Polyanska et al., 2017). In rodents, the mPFC has an important role in executive functions including attention (Fisher et al., 2020) and cognitive flexibility; in terms of set‐shifting (Birrell & Brown, 2000). Thus, *Nrxn1α* ^+/−^ mice may model aspects of executive dysfunction in these disorders, and the PFC may be dysfunctional in humans with 2p16.3 deletions. This certainly warrants further systematic investigation.

We found that mPrL metabolism was negatively correlated with LMA in male but not in female mice. Thus, mPrL hypometabolism may contribute to the hypolocomotor phenotype seen selectively in male *Nrxn1α* ^+/−^ mice. This suggestion is consistent with data showing that mPFC lesions reduce LMA (Jinks & McGregor, 1997) and that modulating GABAergic (Asinof and Paine, 2013) or serotonergic (Takahashi et al., 2000) mPFC neurotransmission influences LMA. The cellular and neurochemical basis of the PFC dysfunction seen in *Nrxn1α* ^+/−^mice requires further characterization. However, we found clear evidence for reduced insulin receptor signaling in the PFC, which likely contributes to the mPrL hypometabolism present in these animals. Importantly, all the aforementioned studies implicating the mPFC in the regulation of LMA were conducted in male rodents. As far as we are aware, whether these manipulations impact on LMA in female rodents has not yet been characterized. Our data suggest that these effects may be sex specific.

mPrL metabolism also correlated with the ability of animals to learn a novel odor discrimination, with lower metabolism associated with better performance (fewer TTC). Therefore, the mPrL hypometabolism seen in *Nrxn1α* ^+/−^ mice may relate to their improved ability for novel odor discrimination. The mPrL has a primary role in discriminating between rewarded and non‐rewarded environmental cues (Fisher et al., 2020; Sangha et al., 2014), supporting the suggestion that the altered mPrL function seen in *Nrxn1α* ^+/−^ mice may contribute to their enhanced performance. As the ^14^C‐2‐deoxyglucose signal largely represents the functional activity of synapses (Nudo & Masterton, 1986), whether the hypometabolism seen in the mPFC reflects altered local GABAergic or glutamatergic synaptic neurotransmission would be of interest.

The DRN hypermetabolism identified in *Nrxn1α* ^+/−^ mice suggests that serotonergic neurotransmission may be altered. To our knowledge, there are currently no additional published data on the impact of *Nrxn1α* heterozygosity on serotonin system function. However, one post‐synaptic binding partner of Nrxn1α, Neuroligin 2 (Nlgn2), impacts on the function of the serotonin system, through direct interaction with the serotonin transporter (Ye et al., 2015). Whether the serotonin system is dysfunctional in *Nrxn1α* ^+/−^ mice certainly warrants further investigation, given the reported alterations in serotonin system function in other relevant rodent models (Guo & Commons, 2016), and in order to understand the potential relevance to serotonin system dysfunction in ASD (Muller et al., 2016) and ScZ (Selvaraj et al., 2014). Interestingly, there was a positive correlation between DRN metabolism and the latency to make correct choices during the initial odor discrimination phase (Discrimination 1). Therefore, the DRN hypermetabolism seen in *Nrxn1α* ^+/−^ mice may relate to their increased correct choice latency. This increased latency suggests that *Nrxn1α* ^+/−^ mice may require more time to make correct choices. The potential role of the DRN in this process is consistent with the observation that serotonin regulates behavioral inhibition and impulsivity (Harrison et al., 1999; Worbe et al., 2014). Interestingly, studies using optogenetic stimulation of DRN serotonergic neurons have identified a key role for these neurons in enhancing patience for receiving a reward, with modeling suggesting that DRN activation increases confidence in the future likelihood of receiving a reward (Miyazaki et al., 2018). In addition, a key role for serotonergic neurotransmission in the mPFC has been identified in this process (Miyazaki et al., 2020). Given our observations, the potential contribution of altered serotonin system function in *Nrxn1α* ^+/−^ mice to correct choice latency, and the potential impact of *Nrxn1α* heterozygosity on impulsive behavior and patience, warrants further investigation. In addition, given that we have identified a potential role for reduced insulin receptor signaling in the reduced glucose metabolism seen in the PFC of *Nrxn1α* ^+/−^ mice, determining whether altered insulin receptor signaling contributes to the increased DR metabolism seen in these animals also would be of interest.

We found no evidence to suggest that metabolism in the mPrL or DRN correlated with reversal learning performance in mice. This is consistent with published data showing that lesioning the mPrL does not impact on reversal learning (Birrell & Brown, 2000). However, published data support a role for the serotonin system in modulating reversal learning, particularly through its actions in the orbitofrontal cortex (Alsio et al., 2021). We found no evidence for altered orbitofrontal cortex function in *Nrxn1α* ^+/−^ mice in this study (Table S2). Further research is needed to understand the neurobiological basis of the reversal learning deficit seen in *Nrxn1α* ^+/−^ mice. However, it is important to state that while correlations between regional activity and behavior can be useful in further understanding the potential neurobiological basis of behavioral deficits in animal models, some deficits are likely to arise from more subtle dysfunction in the interconnectivity of brain networks, and relationships to behavior might not be apparent when regional metabolism is considered. Thus, future studies characterizing the relationship between brain network connectivity and behavioral deficits would be of interest, particularly given the network alterations seen in *Nrxn1a* ^*+/−*^ mice (Hughes et al., 2020).

## CONCLUSIONS

The data suggest that *Nrxn1a* heterozygosity induces alterations in cognition and behavior that are relevant to those seen in ASD and other neurodevelopmental disorders associated with 2p16.3 deletion. *Nrxn1α* heterozygosity alters mPFC and DRN function, which may contribute to some of the behavioral alterations observed. Altered mPFC and serotonin system function may be two key mechanisms through which 2p16.3 deletion impacts on cognition, and by which the CNV increases the risk of developing neurodevelopmental disorders. Targeting the PFC and serotonin system may be useful therapeutic strategies for individuals with 2p16.3 deletion and its' associated neurodevelopmental disorders. Further research is required to more clearly understand the nature of the dysfunction that arises in these systems as a consequence of 2p16.3/*Nrxn1α* heterozygous deletion.

## CONFLICT OF INTEREST

The authors declare that they have no conflict of interest.

## Supporting information


**Figure S1** 

**Figure S2** 

**Figure S3** 

**Supplemental Table S1** Odor pair sets used in the odor‐based discrimination and reversal learning (OB‐DaRL) task

**Supplemental Table S2** (i): Rates of Local Cerebral Glucose Utilization (LCGU) determined in *Nrxn1α* ^*+/−*^ and WT mice – Prefrontal Cortex, Mesolimbic, Cortical, and Basal Ganglia Regions
Supplemental Table S2 (ii): Rates of Local Cerebral Glucose Utilization (LCGU) determined in *Nrxn1α* ^*+/−*^ and WT mice – Septum/DB, Thalamus, Amygdala, Hippocampus, Raphé, and Multimodal Regions

**Supplemental Table S3** Table showing correlations between cerebral metabolism in the mPrL and DRN and open field behaviors in mice.

**Supplemental Table S4.** Table showing correlations between cerebral metabolism in the mPrL and DRN and performance in the odor‐based associative learning and reversal learning (OB‐DaRL) task in mice

**Appendix S1**: Supporting Information

**Appendix S2**: Supporting Information

## Data Availability

The data that support the findings of this study are available from the corresponding author upon reasonable request.
