# Supplementary figures and images for: Altered medial prefrontal cortex and dorsal raphé activity predict genotype and correlate with abnormal learning behavior in a mouse model of autism‐associated 2p16.3 deletion
Source: Autism Res. 2022 Feb 10;15(4):614–27. doi: 10.1002/aur.2685 (PMC9303357; doi:10.1002/aur.2685)

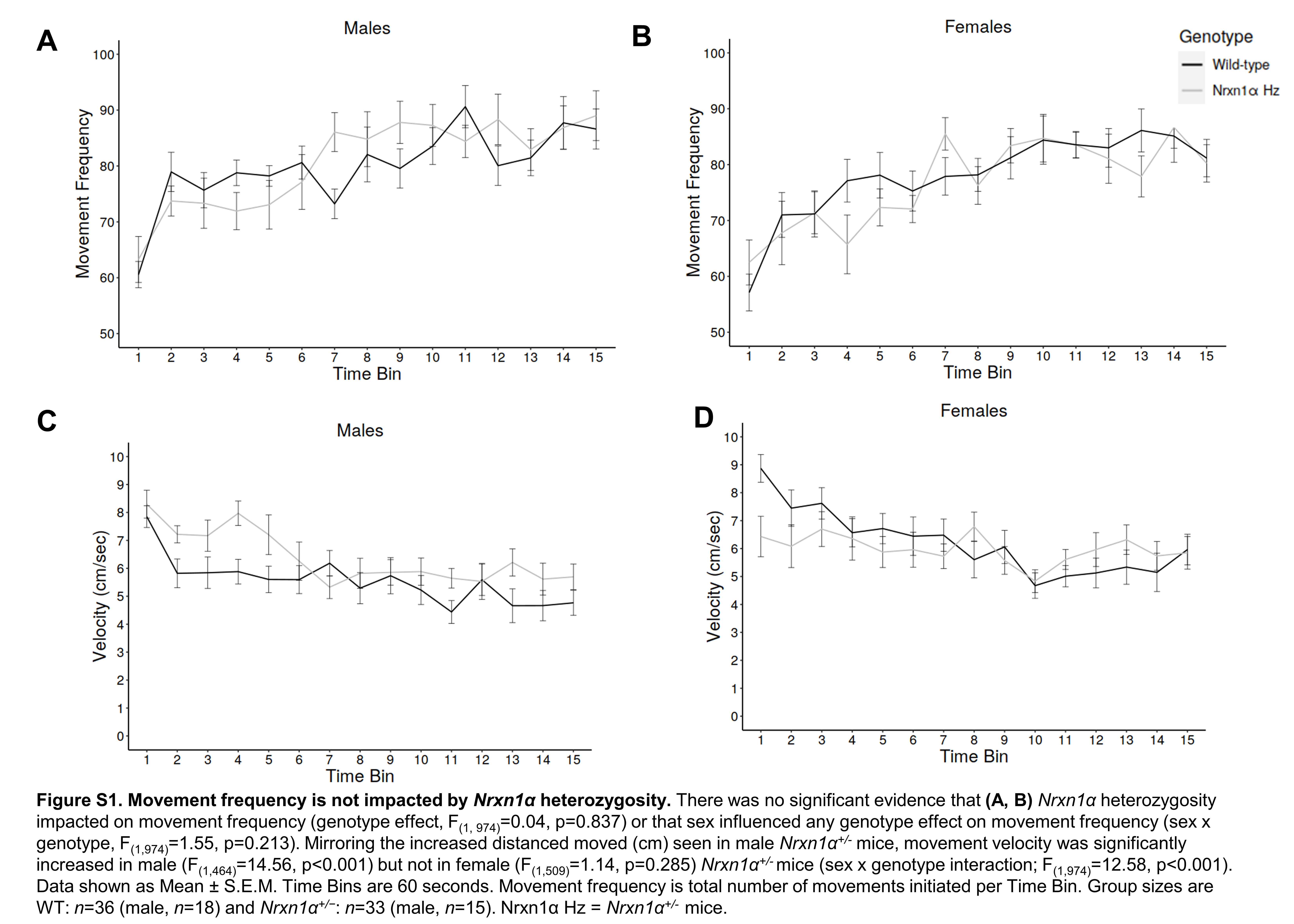

Supplement: Supplementary file 1 — Figure S1 [file AUR-15-614-s003.tif]

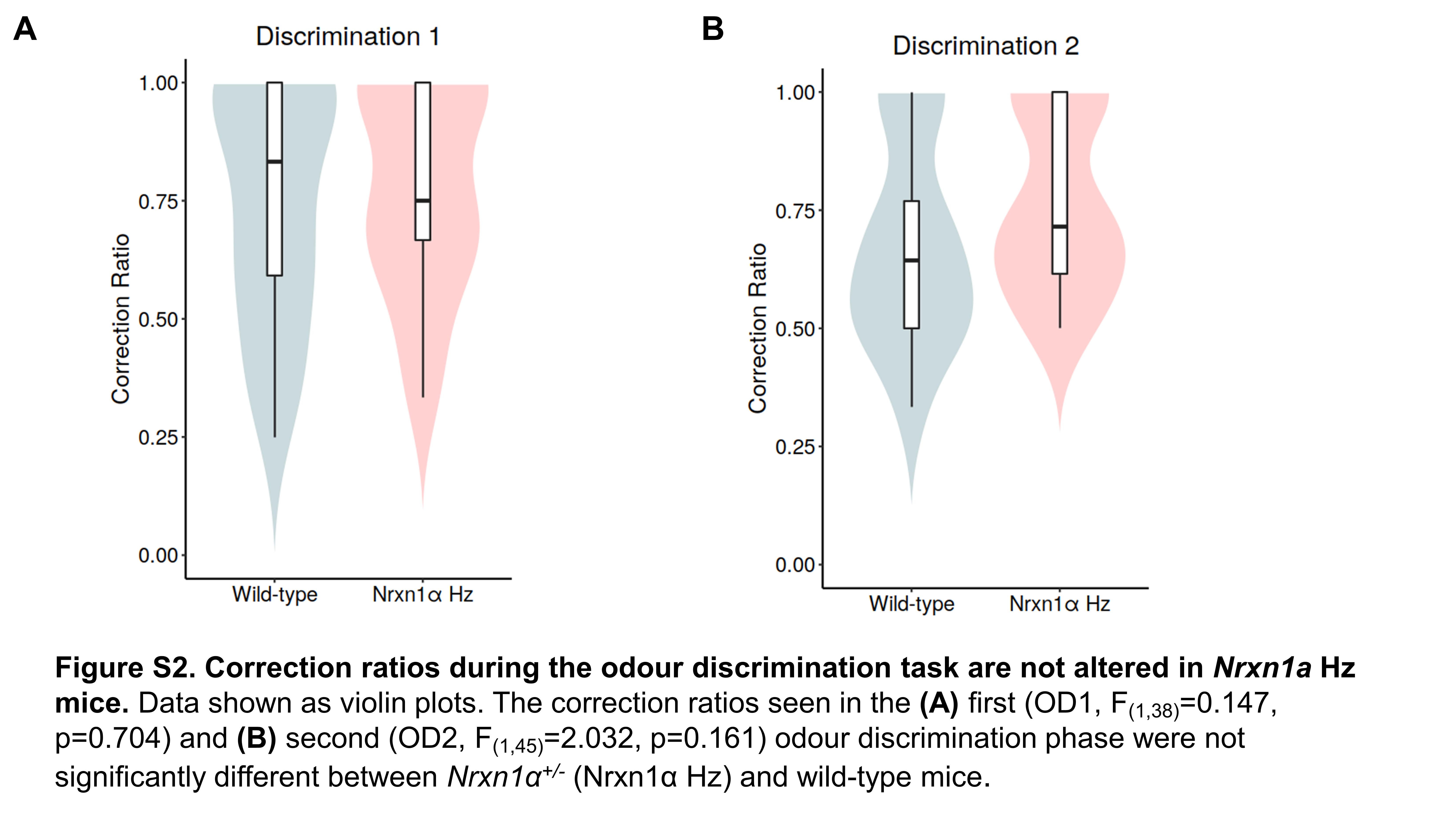

Supplement: Supplementary file 2 — Figure S2 [file AUR-15-614-s004.tif]

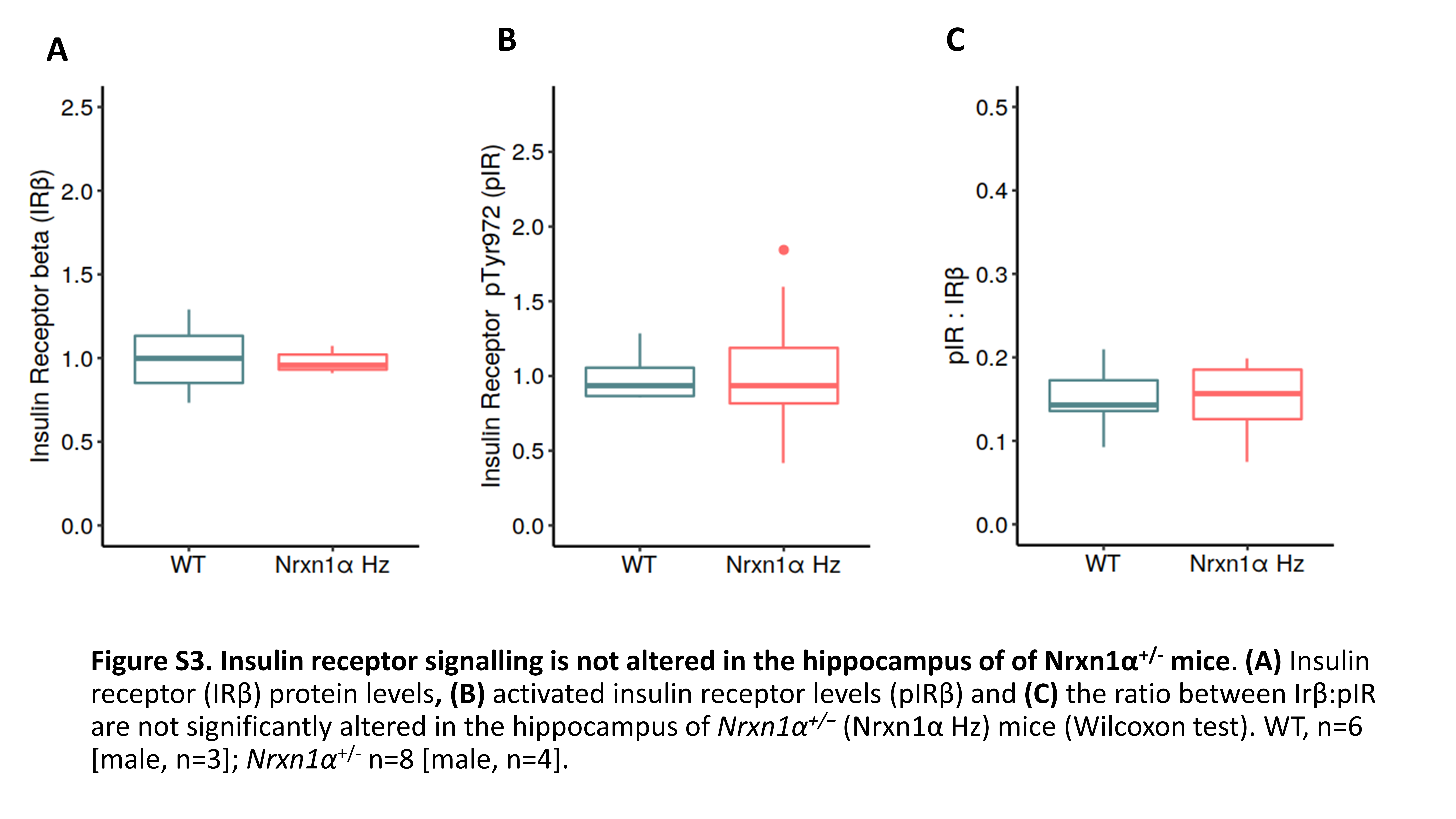

Supplement: Supplementary file 3 — Figure S3 [file AUR-15-614-s001.tif]
