# Supplementary material for: Altered medial prefrontal cortex and dorsal raphé activity predict genotype and correlate with abnormal learning behavior in a mouse model of autism‐associated 2p16.3 deletion
Source: Autism Res. 2022 Feb 10;15(4):614–27. doi: 10.1002/aur.2685 (PMC9303357; doi:10.1002/aur.2685)
Supplement: Supplementary file 4 — Supplemental Table S1 Odor pair sets used in the odor‐based discrimination and reversal learning (OB‐DaRL) task [file AUR-15-614-s006.docx]

**Supplemental Table S1. Odour pair sets used in the odour-based discrimination and reversal learning (OB-DaRL) task**

| **Set 1** | |  | **Set 4** | |
| --- | --- | --- | --- | --- |
| **Task Phase** | **Odour Pair** |  | **Task Phase** | **Odour Pair** |
| **Odour Discrimination 1** | Cumin v Nutmeg |  | **Odour Discrimination 1** | Cumin v Nutmeg |
| **Odour Discrimination 2 and Reversal** | Tumeric v Paprika |  | **Odour Discrimination 2 and Reversal** | Cloves v Cinnamon |
|  |  |  |  |  |
| **Set 2** | |  | **Set 5** | |
| **Task Phase** | **Odour Pair** |  | **Task Phase** | **Odour Pair** |
| **Odour Discrimination 1** | Tumeric v Paprika |  | **Odour Discrimination 1** | Tumeric v Paprika |
| **Odour Discrimination 2 and Reversal** | Cloves v Cinnamon |  | **Odour Discrimination 2 and Reversal** | Cumin v Nutmeg |
|  |  |  |  |  |
| **Set 3** | |  | **Set 3** | |
| **Task Phase** | **Odour Pair** |  | **Task Phase** | **Odour Pair** |
| **Odour Discrimination 1** | Cloves v Cinnamon |  | **Odour Discrimination 1** | Cloves v Cinnamon |
| **Odour Discrimination 2 and Reversal** | Tumeric v Paprika |  | **Odour Discrimination 2 and Reversal** | Cumin v Nutmeg |
