# Supplementary material for: Altered medial prefrontal cortex and dorsal raphé activity predict genotype and correlate with abnormal learning behavior in a mouse model of autism‐associated 2p16.3 deletion
Source: Autism Res. 2022 Feb 10;15(4):614–27. doi: 10.1002/aur.2685 (PMC9303357; doi:10.1002/aur.2685)
Supplement: Supplementary file 5 — Supplemental Table S2 (i): Rates of Local Cerebral Glucose Utilization (LCGU) determined in Nrxn1α +/− and WT mice – Prefrontal Cortex, Mesolimbic, Cortical, and Basal Ganglia Regions Supplemental Table S2 (ii): Rates of Local Cerebral Glucose Utilization (LCGU) determined in Nrxn1α +/− and WT mice – Septum/DB, Thalamus, Amygdala, Hippocampus, Raphé, and Multimodal Regions [file AUR-15-614-s008.docx]

**Supplemental Table S2(i): Rates of Local Cerebral Glucose Utilisation (LCGU) determined in *Nrxn1α^+/-^* and WT mice – Prefrontal Cortex, Mesolimbic, Cortical and Basal Ganglia Regions**

|  | **Wild-type** | | | | | | ***Nrxn1α^+/-^*** | | | | | |
| --- | --- | --- | --- | --- | --- | --- | --- | --- | --- | --- | --- | --- |
|  | **Male** | | | **Female** | | | **Male** | | | **Female** | | |
| **Prefrontal Cortex** |  |  |  |  |  |  |  |  |  |  |  |  |
| anterior Prelimbic Cortex (aPrL) | 1.07 | ± | 0.06 | 1.12 | ± | 0.02 | 1.02 | ± | 0.02 | 1.06 | ± | 0.06 |
| Frontal Association Cortex (FRA) | 1.02 | ± | 0.06 | 1.01 | ± | 0.03 | 1.02 | ± | 0.03 | 1.01 | ± | 0.04 |
| Dorsolateral Orbital Cortex (DLO) | 0.81 | ± | 0.04 | 0.76 | ± | 0.02 | 0.82 | ± | 0.03 | 0.78 | ± | 0.02 |
| Lateral Orbital Cortex (LO) | 1.69 | ± | 0.05 | 1.68 | ± | 0.05 | 1.61 | ± | 0.03 | 1.60 | ± | 0.05 |
| Medial Orbital Cortex (MO) | 0.95 | ± | 0.04 | 0.90 | ± | 0.03 | 0.89 | ± | 0.02 | 0.89 | ± | 0.04 |
| medial Prelimbic Cortex (mPrL) | 1.13 | ± | 0.03 | 1.16 | ± | 0.02 | 1.06** | ± | 0.02 | 1.06** | ± | 0.03 |
| Infralimbic Cortex (IL) | 0.86 | ± | 0.03 | 0.88 | ± | 0.03 | 0.84 | ± | 0.01 | 0.82 | ± | 0.03 |
| **Mesolimbic System** |  |  |  |  |  |  |  |  |  |  |  |  |
| Nucleus Accumbens Core (NaC) | 0.83 | ± | 0.01 | 0.80^+^ | ± | 0.01 | 0.82 | ± | 0.02 | 0.77^+^ | ± | 0.02 |
| Nucleus Accumbens Shell (NaS) | 0.82 | ± | 0.02 | 0.79 | ± | 0.03 | 0.79 | ± | 0.04 | 0.74 | ± | 0.01 |
| Ventral Tegmental Area (VTA) | 0.98 | ± | 0.04 | 1.02 | ± | 0.04 | 1.04 | ± | 0.05 | 1.12 | ± | 0.05 |
| **Cortex** |  |  |  |  |  |  |  |  |  |  |  |  |
| Retrosplenial Cortex (RSC) | 1.47 | ± | 0.05 | 1.45 | ± | 0.06 | 1.52 | ± | 0.04 | 1.51 | ± | 0.04 |
| Piriform Cortex (Piri) | 1.26 | ± | 0.03 | 1.28 | ± | 0.02 | 1.24 | ± | 0.03 | 1.22 | ± | 0.03 |
| Insular Cortex (Ins) | 0.75 | ± | 0.02 | 0.72 | ± | 0.02 | 0.77 | ± | 0.01 | 0.74 | ± | 0.02 |
| Entorhinal Cortex (EntoC) | 0.76 | ± | 0.02 | 0.81 | ± | 0.03 | 0.80 | ± | 0.02 | 0.79 | ± | 0.03 |
| **Basal Ganglia** |  |  |  |  |  |  |  |  |  |  |  |  |
| Dorsolateral Striatum (DLST) | 1.41 | ± | 0.07 | 1.46 | ± | 0.04 | 1.41 | ± | 0.03 | 1.35 | ± | 0.04 |
| Ventromedial Striatum (VMST) | 1.13 | ± | 0.02 | 1.20 | ± | 0.02 | 1.18 | ± | 0.02 | 1.15 | ± | 0.04 |
| Globus Pallidus (GP) | 0.86 | ± | 0.03 | 0.81 | ± | 0.02 | 0.86 | ± | 0.02 | 0.84 | ± | 0.02 |
| Substantia Nigra pars Reticulata (SNR) | 0.73 | ± | 0.05 | 0.67 | ± | 0.03 | 0.70 | ± | 0.03 | 0.72 | ± | 0.02 |
| Substantia Nigra pars Compacta (SNC) | 0.98 | ± | 0.05 | 0.95 | ± | 0.03 | 0.95 | ± | 0.04 | 0.99 | ± | 0.02 |

Data shown as Mean ± SEM. **P<0.01, ***P<0.001 significant difference from wild-type (ANOVA). ^+^P<0.05 significant effect of sex (ANOVA).

**Supplemental Table S2(ii): Rates of Local Cerebral Glucose Utilisation (LCGU) determined in *Nrxn1α^+/-^* and WT mice – Septum/DB, Thalamus, Amygdala, Hippocampus, Raphé and Multimodal Regions**

|  | **Wild-type** | | | | | | ***Nrxn1α^+/-^*** | | | | | |
| --- | --- | --- | --- | --- | --- | --- | --- | --- | --- | --- | --- | --- |
|  | **Male** | | | **Female** | | | **Male** | | | **Female** | | |
| **Septum/DB** |  |  |  |  |  |  |  |  |  |  |  |  |
| Medial Septum (MS) | 0.98 | ± | 0.02 | 0.99 | ± | 0.01 | 0.99 | ± | 0.02 | 0.95 | ± | 0.05 |
| Lateral Septum (LS) | 0.87 | ± | 0.02 | 0.89 | ± | 0.02 | 0.88 | ± | 0.01 | 0.84 | ± | 0.04 |
| Ventral limb of the Diagonal band of Broca (VDB) | 0.95 | ± | 0.02 | 0.97 | ± | 0.03 | 0.94 | ± | 0.03 | 0.89 | ± | 0.05 |
| Horizontal limb of the Diagonal band of Broca (HDB) | 1.03 | ± | 0.01 | 1.04 | ± | 0.02 | 1.01 | ± | 0.02 | 0.98 | ± | 0.03 |
| **Thalamus** |  |  |  |  |  |  |  |  |  |  |  |  |
| Anteromedial Thalamus (AM) | 1.42 | ± | 0.08 | 1.49 | ± | 0.05 | 1.45 | ± | 0.03 | 1.47 | ± | 0.04 |
| Reticular Thalamus (Rt) | 1.54 | ± | 0.07 | 1.50 | ± | 0.07 | 1.44 | ± | 0.05 | 1.51 | ± | 0.04 |
| Mediodorsal Thalamus (MD) | 1.47 | ± | 0.06 | 1.38 | ± | 0.05 | 1.42 | ± | 0.03 | 1.49 | ± | 0.03 |
| Ventrolateral Thalamus (VL) | 1.43 | ± | 0.05 | 1.36 | ± | 0.06 | 1.37 | ± | 0.04 | 1.48 | ± | 0.02 |
| Ventromedial Thalamus (VM) | 1.48 | ± | 0.06 | 1.38 | ± | 0.05 | 1.40 | ± | 0.03 | 1.43 | ± | 0.03 |
| **Amygdala** |  |  |  |  |  |  |  |  |  |  |  |  |
| Basolateral Amygdala (BLA) | 0.86 | ± | 0.02 | 0.83 | ± | 0.03 | 0.76 | ± | 0.03 | 0.82 | ± | 0.04 |
| Central Amygdala (CeA) | 0.50 | ± | 0.02 | 0.44 | ± | 0.02 | 0.46 | ± | 0.03 | 0.49 | ± | 0.03 |
| Medial Amygdala (MeA) | 0.59 | ± | 0.04 | 0.54 | ± | 0.03 | 0.56 | ± | 0.03 | 0.57 | ± | 0.04 |
| **Hippocampus** |  |  |  |  |  |  |  |  |  |  |  |  |
| Molecular Layer (LMol) | 1.03 | ± | 0.03 | 1.09 | ± | 0.04 | 1.05 | ± | 0.02 | 1.06 | ± | 0.02 |
| Cornu Ammonis 1 (CA1) | 0.76 | ± | 0.03 | 0.78 | ± | 0.02 | 0.75 | ± | 0.03 | 0.82 | ± | 0.04 |
| Cornu Ammonis 3 (CA3) | 0.67 | ± | 0.02 | 0.68 | ± | 0.02 | 0.66 | ± | 0.03 | 0.68 | ± | 0.03 |
| Dentate Gyrus (DG) | 0.78 | ± | 0.03 | 0.81 | ± | 0.03 | 0.79 | ± | 0.02 | 0.82 | ± | 0.03 |
| **Raphé** |  |  |  |  |  |  |  |  |  |  |  |  |
| Dorsal Raphé (DRN) | 0.77 | ± | 0.04 | 0.73 | ± | 0.03 | 0.85*** | ± | 0.02 | 0.83*** | ± | 0.02 |
| Median Raphé (MRN) | 1.18 | ± | 0.04 | 1.21^+^ | ± | 0.03 | 1.15 | ± | 0.02 | 1.27^+^ | ± | 0.03 |
| **Multimodal** |  |  |  |  |  |  |  |  |  |  |  |  |
| Mamillary Body (MB) | 1.74 | ± | 0.06 | 1.86 | ± | 0.13 | 1.79 | ± | 0.04 | 1.74 | ± | 0.05 |
| Medial Geniculate (MG) | 1.28 | ± | 0.05 | 1.36 | ± | 0.05 | 1.34 | ± | 0.03 | 1.37 | ± | 0.04 |

Data shown as Mean SEM. **P<0.01, ***P<0.001 significant difference from wild-type (ANOVA). ^+^P<0.05 significant effect of sex (ANOVA).
