# Supplementary material for: Altered medial prefrontal cortex and dorsal raphé activity predict genotype and correlate with abnormal learning behavior in a mouse model of autism‐associated 2p16.3 deletion
Source: Autism Res. 2022 Feb 10;15(4):614–27. doi: 10.1002/aur.2685 (PMC9303357; doi:10.1002/aur.2685)
Supplement: Supplementary file 6 — Supplemental Table S3 Table showing correlations between cerebral metabolism in the mPrL and DRN and open field behaviors in mice. [file AUR-15-614-s002.docx]

**Table S3. Table showing correlations between cerebral metabolism in the mPrL and DRN and open field behaviours in mice**

|  | **All mice** | | **Male mice** | | **Female mice** | |
| --- | --- | --- | --- | --- | --- | --- |
|  |  | |  | |  | |
|  | **r** | **p-value** | **r** | **p-value** | **r** | **p-value** |
| **Medial Prelimbic Cortex (mPrL)** | | | | | | |
| Average Velocity (cm/s) | -0.259 | 0.127 | **-0.490** | **0.032** | -0.123 | 0.637 |
| Total Distance Moved (cm) | -0.255 | 0.134 | **-0.491** | **0.033** | -0.130 | 0.618 |
| Movement duration | -0.225 | 0.187 | **-0.469** | **0.043** | -0.060 | 0.820 |
| Central zone duration (seconds) | 0.147 | 0.391 | -0.063 | 0.798 | 0.275 | 0.286 |
| **Dorsal Raphé (DRN)** | | | | | | |
| Average Velocity (cm/s) | -0.011 | 0.948 | -0.213 | 0.380 | 0.318 | 0.214 |
| Total Distance Moved (cm) | -0.013 | 0.9393 | -0.212 | 0.383 | 0.312 | 0.222 |
| Movement duration (secs) | -0.040 | 0.818 | -0.269 | 0.265 | 0.329 | 0.197 |
| Central zone duration (secs) | 0.004 | 0.978 | -0.143 | 0.558 | 0.142 | 0.59 |

**Table S4. Table showing correlations between cerebral metabolism in the mPrL and DRN and performance in the odour-based associative learning and reversal learning (OB-DaRL) task in mic**e

|  | **Medial Prelimbic Cortex (mPrL)** | | **Dorsal Raphé (DRN)** | |
| --- | --- | --- | --- | --- |
|  | **r** | **p-value** | **r** | **p-value** |
| **Training Discrimination (OD1)** | | | | |
| Trials to Criteria (TTC) | **0.486** | **0.007** | -0.219 | 0.255 |
| Percentage Correct | -0.076 | 0.695 | 0.196 | 0.308 |
| Correct Choice Response Latency (secs) | -0.094 | 0.602 | **0.379** | **0.030** |
| **Reversal Learning (ODR)** | | | | |
| Trials to Criteria (TTC) | -0.238 | 0.2218 | -0.041 | 0.835 |
| Percentage Correct | 0.314 | 0.091 | -0.191 | 0.312 |
|  |  |  |  |  |
|  |  |  |  |  |
