# Supplementary material for: Altered medial prefrontal cortex and dorsal raphé activity predict genotype and correlate with abnormal learning behavior in a mouse model of autism‐associated 2p16.3 deletion
Source: Autism Res. 2022 Feb 10;15(4):614–27. doi: 10.1002/aur.2685 (PMC9303357; doi:10.1002/aur.2685)
Supplement: Supplementary file 7 — Appendix S1: Supporting Information [file AUR-15-614-s007.docx]

**Decision Tree Classification of *Nrxn1α* Genotype using ^14^C-2-Deoxyglucose Functional Brain Imaging Data**

Decision tree classifiers have been used widely in many previous studies for classifying biological data, including in the classification of patients on the basis of brain imaging data (Mudali et al., 2015, Stolicyn et al., 2020). The decision tree classifier method is a supervised machine learning approach that builds a classifier from a set of training samples with a list of known features and the class label. In terms of our data set, the class label is whether the animal is wild-type (WT) or *Nrxn1α* heterozygous (*Nrxn1α^+/-^*) and the features included in the model are the rate of cerebral metabolism (as reflected by the ^14^C-2-deoxyglucose (^14^C-2-DG) uptake ratio) in distinct brain regions. The algorithm sequentially splits the data into subsets, with the data in the descending subset gained being purer in terms of the classification of the sample than that of the parent set (i.e. the new subset contains a greater proportion of classification than the parent set). Each split is based on the optimal threshold value of a single feature (metabolism in a given brain region) and is based on the feature and threshold that yields the largest information gain at each node. These classificaitons are made in a stepwise manner. The decision tree constructed using the training data can be then be used to predict the classification of previously unseen cases, where the class identity is unknown. The decision trees also provide information on which features (brain regions) are most important in terms of the classification, and provides threshold values for each feature that the algorithm has used to separate the samples into the non-overlapping classifications. In the context of our study, this is useful in further understanding which brain regions are dysfunctional in *Nrxn1α^+/-^* mice, and the rates of metabolism in those regions that are predictive of *Nrxn1α* heterozygosity.

In this study we used the Decision Tree Classifier in the python module Scikit learn (<https://www.scikit-learn.org>), which employs the Classification and Regression Trees (CART) algorithm. As a supervised machine learning technique, the application of Decision Tree Classification to training data requires testing and optimisation to determine the model parameters that are most effective in generating a decision tree that has optimal performance in terms of classification accuracy, sensitivity and specificity. Accuracy is the ability to classify a data point correctly, sensitivity (true positive rate) is the ability of the algorithm to correctly classify positive cases (e.g. *Nrxn1α^+/-^* mice correctly) and specificity (true negative rate) is the ability of the algorithm to correctly classify negative cases (e.g. WT mice) correctly. Accuracy, sensitivity and specificity are formally defined as:

**Accuracy =**

number of correct predictions (TP + TN) / total number of predictions (TP + TN + FP + FN),

**Sensitivity =**

number of correct true case predictions (TP) / total number of true cases (TP + FN),

**Specificity =**

number of correct negative case predictions (TN) / total number of negative cases (TN + FP),

where TP = true positive, TN = true negative, FN = false negative, FP = false positive

It is important to generate a decision tree that has these predictive attributes, but also one that is generalizable and that does not over-fit the cases included in the training data set. For example, while it would be possible to generate a decision tree classifier that produces 100% accuracy in the classification of the training data, it may over-fit the training data and result in poor performance in the classification of data generated outside of the training data set. Thus, it is important to “prune” decision trees (i.e. limit their depth in terms of the number of features they use in their classification) to ensure that the data do not over-fit the training data, and that the decision tree is generalizable to relevant data outside of the training data.

To establish the algorithm and tree depth that gives optimal performance, in terms of classifying *Nrxn1α* genotype on the basis of the ^14^C-2-DG functional brain imaging data, we characterised CART decision trees that utilised two different criterion for measuring the quality of each split in the decision tree; the Gini criterion for impurity and the entropy criterion for information gain. In addition, to determine the optimal tree depth, we first characterised the performance of un-pruned trees on the data set (which gave trees with a depth of 4 or 5) and then characterised the properties of pruned trees of lower depth (2 or 3 layers).

To determine the decision tree parameters that resulted in optimal tree structure and classification performance we first characterised the ability of the decision trees to classify unseen data (in terms of accuracy, sensitivity and specificity) using a leave-one-out cross validation (LOOCV) strategy. In LOOCV a single observation from the training data set is removed, to be used as a test set for later classification, and the remaining observations are used to generate the decision tree classifier. The decision tree is generated, and the ability of the decision tree to correctly classify the removed case tested. This procedure is repeated *N* times, with each observation used once as a test (validation) set. Table 1 shows the resulting performance of the decision trees classifiers tested through this process.

**Optimal decision tree parameters for *Nrxn1α* genotype classification on the basis of cerebral metabolism**

LOOCV indicated that the decision trees offering optimal classification performance were those that utilised classification on the basis of the Gini (impurity) index, which showed better performance overall than when the entropy (information gain) criterion was used (Table 1). For decision trees employing the entropy classification, optimal performance was found for unpruned trees (that had a depth of 4 or 5). These gave an accuracy of 67%, a specificity of 58% and a sensitivity of 75%. When the Gini criterion was employed, unpruned trees (that had a depth of 4 or 5) performed slight better than those using the entropy classification, having an accuracy of 69%, a specificity of 63% and a sensitivity of 75%. However, decision trees generated using the Gini criterion that were pruned to a maximum depth of 2 offered optimal performance during LOOCV, with an accuracy of 79%, a specificity of 68% and a sensitivity of 90%. Thus, the decision trees generated using these parameters were viewed as being most informative in terms of differentiating between *Nrxn1α^+/-^* and WT mice. These trees also provided important information on which features (brain regional metabolism) is most useful for differentiating between the two genotypes.

|  | **Entropy - Information Gain** | | | | **Gini – Impurity** | | | |
| --- | --- | --- | --- | --- | --- | --- | --- | --- |
| Max Depth | **No Pruning** | **4** | **3** | **2** | **No Pruning** | **4** | **3** | **2** |
| Accuracy | 0.67 | 0.62 | 0.64 | 0.56 | 0.69 | 0.74 | 0.72 | **0.79** |
| Sensitivity | 0.75 | 0.71 | 0.71 | 0.71 | 0.75 | 0.86 | 0.8 | **0.9** |
| Specificity | 0.58 | 0.5 | 0.56 | 0.39 | 0.63 | 0.61 | 0.63 | **0.68** |

**Table 1. Decision tree classifier performance for the classification of *Nrxn1α^+/-^* from WT mice on the basis of regional cerebral metabolism.** Performance was assessed by LOOCV, with values closer to 1.00 (100%) indicating better performance. Decision trees were generated using either the Entropy (information gain) or Gini (impurity) criterion. Decision trees were either unpruned (automatically generating trees with a depth of 4 or 5) or pruned by maximum depth (2-4). LOOCV identified trees generated using the Gini index with a maximum depth of 2 as having optimal performance for the classification of *Nrxn1α^+/-^* mice on the basis of cerebral metabolism.

**Regional cerebral metabolism important in the decision tree classification of *Nrxn1a^+/-^* mice**

By looking at the features identified as being important in the training decision trees generated through our LOOCV analysis we are able to identify the features, in terms of the regional cerebral metabolism, that are most useful in differentiating *Nrxn1α^+/-^* from WT mice. Therefore, we characterised the brain regions most frequently seen in these LOOCV training decision trees and the depth at which these regions were most frequently seen (Table 2). Those regions nearer the root of the tree (level 1) have greater utility in the classification, and are relevant to more observations than those at lower levels. Given the optimal performance of decision trees using the Gini criterion with a maximum depth of 2 (Table 1), we used these trees to identify the regional metabolism important in differentiating between *Nrxn1α^+/-^* and WT mice.

Aligned with our observations that cerebral metabolism was significantly decreased in the mPrL and increased in the DRN of *Nrxn1α^+/-^* mice (Figure 3), both regions were identified as being important contributors to the decision trees generated in the LOOCV analysis, with the mPrL being present in 87% of the decision trees generated and the DRN being present in 31%.

The mPrL was identified as most frequently being the root (level 1 node) of the decision tree, being the first node in 69% of the trees generated. This indicates the importance of metabolism in this region in differentiating between *Nrxn1α^+/-^* and WT mice. In the mPrL, metabolism <=1.153 or <=1.081 differentiated *Nrxn1α^+/-^* from WT mice, depending on the decision tree generated (see Figure 1 below). The DRN was most commonly seen at level 2 in the decision tress, being seen in 18% of decision trees at that level, with metabolism <=0.87 or <=0.728 used to differentiate WT from *Nrxn1α^+/-^* HZ mice, depending on the training decision tree generated (see Figure 1 below).

Interestingly, the Nucleus Accumbens Shell (NaS) was also present in a high proportion of the LOOCV decision trees generated (85%) being most frequently seen at level 2 (67% of the decision trees generated). In this case metabolism being <=0.773 was used to classify WT from *Nrxn1α^+/-^* mice. At the group level cerebral metabolism was not significantly altered in the NaS in *Nrxn1α^+/-^* mice (F_(1,31)_=2.817, p=0.103). However, the inclusion of this region in the decision trees indicates that metabolism in this region may be useful for differentiating a subset of *Nrxn1α^+/-^* from WT mice, once other factors, such as metabolism in the mPFC or DRN have been taken into account. This suggestion is consistent with the observation that the NaS is most commonly seen at level 2 of the decision tree.

The VH-CA3 subfield was also identified as being present in high proportion of LOOCV decision trees, being seen in 21% of all models generated with all of these being seen at level 2 of the decision tree. In this case cerebral metabolism in the VH-CA3 region being <=0.75 was useful in differentiating WT from *Nrxn1α^+/-^* mice (Figure 1). Again, at the group level, cerebral metabolism in the VH-CA3 was not significantly between *Nrxn1α^+/-^* and WT mice (F_(1,31)_=0.021, p=0.886), indicating that metabolism in this region was useful in differentiating a subset of *Nrxn1α^+/-^* mice from WT animals when other features, such as metabolism in the mPrL, had been taken into account. This is consistent with the observation that this region is only seen at level 2 of the decision trees.

There were a number of other regions that were present in a small proportion (<=10%) of the decision trees generated by LOOCV, with all of these features being present at level 2. This included other hippocampal subfields (VL-MoL), multiple regions of the septum/diagonal band of broca system (MS, LS, HDB), the retrosplenial cortex (RSC) and the basolateral amygdala (BLA). Given that these regions are seen in a small proportion of the LOOCV decision trees generated, and their positioning at level 2 in all cases, while these may be useful in differentiating between a small number of *Nrxn1α^+/-^* and WT mice, once metabolism in other brain regions has been taken into account, the wider generalisability of these regions in differentiating between WT and *Nrxn1α^+/-^* mice may be limited.

Overall, these data indicate that the decision tree algorithm can be effectively applied to functional brain imaging data to differentiate between *Nrxn1α^+/-^* and WT mice, with an optimal performance of 79% accuracy, 68% specificity and a 90% sensitivity assessed by LOOCV. Regions useful in this classification include those found to be significantly different between the two genotypes at the group level, including the mPrL and DRN, and other regions that allow the classification of subsets of *Nrxn1α^+/-^* mice once metabolism in the mPrL/DRN has been accounted for. In this case, regional metabolism in the NaS and VH-CA1 may be particularly useful. Representative decision trees generated during LOOCV analysis are shown in Figure 1.

| **Region** | **Total** | **Level 1** | **Level 2** | **Most Frequent Depth** |
| --- | --- | --- | --- | --- |
| mPrl | 87% | 69% | 18% | 1 |
| NaS | 85% | 18% | 67% | 2 |
| DR | 31% | 13% | 18% | 2 |
| VH-CA3 | 21% | 0 | 21% | 2 |
| VMST | 10% | 0 | 10% | 2 |
| RSC | 10% | 0 | 10% | 2 |
| MS | 10% | 0 | 10% | 2 |
| HDB | 10% | 0 | 10% | 2 |
| LS | 8% | 0 | 8% | 2 |
| BLA | 8% | 0 | 8% | 2 |
| VL-Mol | 5% | 0 | 5% | 2 |

**Table 2. Table showing the regions identified in LOOCV decision trees classifying *Nrxn1α^+/-^* mice on the basis of cerebral metabolism.** LOOCV decision trees were generated using the Gini criterion with a maximum depth of 2. Data show the total percentage of decision trees each region was found in, along with the percentage of times each region was seen at level 1 and 2 of all the LOOCV decision trees generated. The most frequent node depth for each region is also reported.


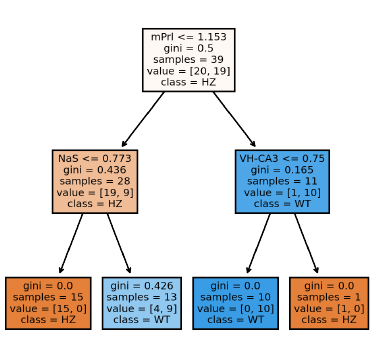

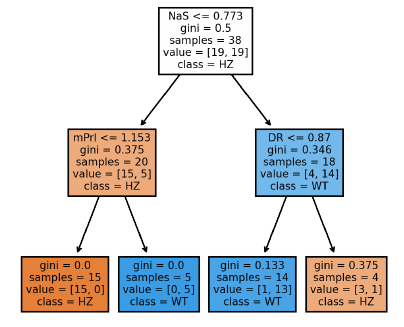


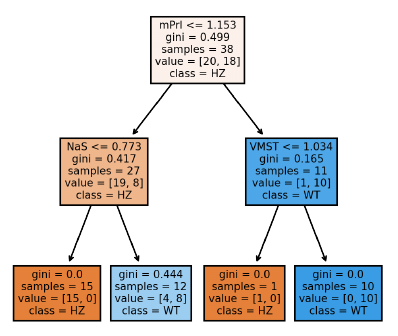

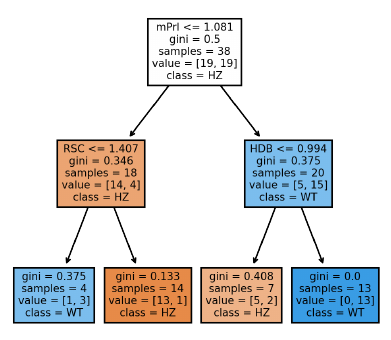


**Figure 1. Representative decision tree structures for classification of *Nrxn1α^+/-^* and WT mice on the basis of regional cerebral metabolism.** **(A)** Decision tree structure generated from the whole training data set with no observations excluded. This was also the most commonly seen decision tree structure seen across the LOOCV models generated (present in 21% of trees). **(B)** The second most commonly seen decision tree structure across the LOOCV models generated (18% of models). (**C and D**) The joint third most common decision tree structures seen across the LOOCV models (each 10% of models generated). The value of regional metabolism used for classification is shown at each level. If this value is met then cases are moved to the left node, or else they are moved to the right node. Gini indicates the gini index, reflecting sample purity at each node (minimum=0.5: balanced mixture of classifications, maximum=0: pure classification of one category). The number of individuals present at each level is also shown, with the broad classification of the samples present in that node (“class”) also indicated. Value indicates the number of observations for each classification type (WT or *Nrxn1α^+/-^*) present at each level, with *Nrxn1α^+/-^* samples indicated by the left and WT mice indicated by the right integer.

56% of the Decision trees generated during LOOCV involved the mPrL at level 1 and the NaS at level 2 (Figure 1A and 1C). In these models the mPrL at level 1 differentiated mice into a group that was heavily WT (1 HZ to 10 WT, gini=0.165) and a group that was predominantly *Nrxn1α^+/-^* (19 HZ, 9 WT, gini=0.436). The predominantly Hz group were then classified by metabolism in the NaS into a fully HZ group (15 HZ to 0 WT, gini=0) and one that was predominantly WT (4 HZ to 9 WT). Thus classification on the basis of metabolism in the mPrL being <=1.1553 at the root (level 1) and NaS metabolism being <=0.773 at level 2 performed the vast majority of the classification in the training data sets. The region used to further classify the heavily WT group at level 2 (1 Hz to 10WT) varied across the LOOCV decision trees generated, which is to be expected when classifying only one HZ animal from WT, but commonly involved either the VH-CA3, VMST, MS, LS, BLA in the LOOCV decision trees. The generalisability of any node for characterising *Nrxn1α^+/-^* Hz from WT mice is likely to be small when it is responsible for separating a small number of observations within the training data (e.g. VH-CA3, separating 1 HZ from 10 WT mice at level 2, Figure 1A).

The other type of decision tree that was most commonly generated (18%) in the LOOCV analysis involved the NaS at level 1 and the mPrL at level 2, with metabolism <=0.773 and <=1.153 indicating *Nrxn1α^+/-^* genotype, respectively. In all decision trees generated with this structure the DR was identified as a level 2 node (Figure 1B) with metabolism <=0.87 allowing the classification of WT mice.

The final most common decision tree structure seen (13%) in LOOCV involved the DR at level 1 with metabolism <=0.728 indicating WT genotype. In most of these trees (60%) the NaS differentiated between WT and *Nrxn1α^+/-^* mice at level 2, with the VH-CA3 being included at level 2 in the remaining (40%) of decision trees.

**Validating the predictive ability of the LOOCV decision trees against an externally generated test set**

To further test the predictive validity of the decision trees generated by LOOCV of the training data, in classifying *Nrxn1α^+/-^* and WT genotype on the basis of regional cerebral metabolism, we tested the ability of each of the model trees generated to correctly classify mice from an independently generated test set. This test set involved 8 WT (male n= 4, female n=4) and 6 *Nrxn1α^+/-^* (male n=3, female n=3) mice. The performance of the decision trees in classifying these animals was assessed in terms of accuracy, specificity and precision. Precision is the ability of the classifier not to mislabel a given case; precision = TP / (TP + FP). We also calculated the F-beta score, the weighted harmonic mean of precision and recall, with 1.00 being the optimal value and 0 being the worst.

Table 3 summarises model performance for the decision trees employed in classifying this independent test data. Models involving the mPrL and NaS (at level 1 and 2, respectively), with or without an additional region for classification at level 2, had an accuracy of 79-93% in classifying animals in this independent test set, along with a precision of 71-83% and a specificity of 83-100% (Table 3). Models involving the DRN for classification at the root level (level 1) yielded similar results. When the NaS was included at level 2 in these decision trees accuracy was 93%, precision was 100% and specificity was 89%. A lower level of performance was seen when the decision tree had the VH-Lmol at level 2, with 71% accuracy, 83% precision and 62% specificity. These data prove the utility and generalisability of decision trees involving the mPrL, NaS and DR in accurately classifying *Nrxn1α* genotype on the basis of cerebral metabolism in these regions.

The final LOOCV model generated was one involving the mPrL at level one, and the RSC and HDB at level too. This decision tree model gave poor performance, with an accuracy of 36%, a precision of 29% and a sensitivity of 33% for classifying *Nrxn1α^+/-^* mice from the test validation set. This suggest that this decision tree model has little utility in classifying *Nrxn1α^+/-^* mice on the basis of metabolism in these regions, and this is reflected in the relatively small number of decision tree generated during LOOCV with this structure (10%). This suggests that this tree structure is not generalizable to the classification of mice out with the training data set.

|  | **mPrL and NaS models** | | | | | | | **DR models** | | **mPrL model** |
| --- | --- | --- | --- | --- | --- | --- | --- | --- | --- | --- |
| **Model number** | **1** | **2** | **3** | **4** | **5** | **6** | **7** | **8** | **9** | **10** |
| **Level 1** | **mPrL**  (Hz<=1.153) | **mPrL**  (Hz<=1.153) | **mPrL**  (Hz<=1.153) | **mPrL**  (Hz<=1.153) | **mPrL**  (Hz<=1.153) | **mPrL** (Hz<=1.153) | **NaS** (Hz<=0.773) | **DR**  (WT<=0.728) | **DR** (WT<=0.728) | **mPrL** (Hz<=1.081) |
| **Level 2** | **NaS**  (Hz<=0.773) | **NaS**  (Hz<=0.773) | **NaS**  (Hz<=0.773) | **NaS**  (Hz<=0.773) | **NaS**  (Hz<=0.773) | **NaS** (Hz<=0.773) | **mPrL** (Hz<=1.153) | **VH-Lmol** (Hz<=1.148) | **NaS** (Hz<=0.791) | **RSC** (WT<=1.407) |
| **Level 2** | **MS**  (Hz<=0.898) | **VMST**  (Hz<=1.034) | **LS**  (Hz<=0.831) | **BLA**  (WT<=0.979) | **VH-CA3**  (Hz<=0.75) | **-** | **DR**  (Hz<=0.87) | **-** | **-** | **HDB** (Hz<=0.994) |
| **Accuracy** | 86% | 86% | 93% | 86% | 86% | 86% | 79% | 71% | 93% | 36% |
| **Precision:** *Nrxn1α^+/-^* | 83% | 83% | 86% | 83% | 83% | 83% | 71% | 62% | 100% | 29% |
| **Precision:**  WT | 88% | 88% | 100% | 88% | 88% | 88% | 86% | 83% | 83% | 43% |
| **Specificity:** *Nrxn1α^+/-^* | 83% | 83% | 100% | 83% | 83% | 83% | 83% | 83% | 89% | 33% |
| **Specificity:**  WT | 88% | 88% | 88% | 88% | 88% | 88% | 75% | 62% | 100% | 38% |
| **F-beta score:** *Nrxn1α^+/-^* | 0.83 | 0.83 | 0.92 | 0.83 | 0.83 | 0.83 | 0.77 | 0.71 | 0.91 | 0.31 |
| **F-beta score:**  WT | 0.88 | 0.88 | 0.93 | 0.88 | 0.83 | 0.88 | 0.8 | 0.71 | 0.94 | 0.4 |

**Table 3. Decision tree classifier performance in classifying *Nrxn1α^+/-^* genotype on the basis of regional cerebral metabolism in animals from an externally generated test set.** Decision tree models tested were generated from LOOCV on the training data set, and broadly identified decision trees including the medial prelimbic cortex (mPrL) and nucleus accumbens shell (NaS), or trees involving the dorsal raphé (DRN), as useful in predicting *Nrxn1α^+/^* genotype. Performance of the decision tree classifiers was assessed using an externally generated test set of mice (WT n=6, *Nrxn1α^+/-^* n=8) with performance assessed on the basis of accuracy, precision and specificity. The F-beta score is also shown. Decision tree models that include the mPrL at level 1 and NaS at level 2, or the DRN at level 1 and NaS at level 2, offer the best classification performance.

**References**

Mudali D., Teune L.K., Renken R.J., Leenders K.L., Roerdink J.B. (2015). Classification of Parkinsonian syndromes from FDG-PET brain data using decision trees with SSM/PCA features. *Comput Math Methods Med*, 2015, 136921. <https://doi.org/10.1155/2015/136921>.

Stolicyn A., Harris M.A., Shen X., Barbu M.C., Adams M.J., Hawkins E.L., *et al*. (2020). Automated classification of depression from structural brain measures across two independent community-based cohorts. *Hum Brain Mapp*, 41, 3922-37. <https://doi.org/10.1002/hbm.25095>.
