# Supplementary material for: Altered medial prefrontal cortex and dorsal raphé activity predict genotype and correlate with abnormal learning behavior in a mouse model of autism‐associated 2p16.3 deletion
Source: Autism Res. 2022 Feb 10;15(4):614–27. doi: 10.1002/aur.2685 (PMC9303357; doi:10.1002/aur.2685)
Supplement: Supplementary file 8 — Appendix S2: Supporting Information [file AUR-15-614-s005.docx]

**Supplemental Information – Methods**

1. **Odour-based Discrimination and Reversal Learning (OB-DaRL) Task**

The apparatus consisted of a modified home cage (length 36cm, height 14cm, width 20cm) partitioned into one large holding area (23cm x 20cm) and two smaller “choice” areas (12cm x 10cm). Access to the “choice” areas was restricted by the experimenter using translucent red Perspex doors. A large door restricted access to both choice areas, and smaller dividers were used to restrict access to only one of the “choice” areas, when required. A translucent red Perspex lid covered the home cage. Animals had access to water throughout the task from a small bowl (2cm diameter, 1cm height) contained within the holding area. Two glass bowls (5cm diameter, 3.5cm height) containing the scented digging media were presented in the “choice” areas. The bowls were part-filled (~1 cm depth) with the digging media (autoclaved playground sand) scented with spices (turmeric, paprika, cloves, nutmeg, cinnamon, cumin; Schwartz, UK) which concealed the food pellet reward. 100g of sand contained 0.1g of spice (odor cue) and 0.01g of crushed food reward, to prevent animals from potentially using the scent of the food reward to guide their behaviour. During training and testing the odor pairs were presented as shown in the supplementary material (Table S1), with the odor pair sequences used counterbalanced across the experimental groups and randomly allocated (see supplementary Table S1 for all possible sequences). The OB-DaRL task testing protocol took place over three consecutive days, with two apparatus/odor habituation sessions (days 1 and 2), an initial training odor discrimination (day 2) and a second odor discrimination and the reversal learning test phase (day 3). The procedures for the OR-DaRL task were adapted from similar approaches employed in published work in rats (Birrell and Brown, 2000; Dawson et al., 2012) and mice (Young et al., 2010; Tanaka et al., 2011).

*1.1 Habituation and Learning to Dig*

Prior to the first test day, animals were habituated to the glass bowls and the reward sucrose pellets by placing these into the animal's home cage, for 4 days. During the first test day (training day 1) animals were habituated to the apparatus and were trained to dig in the bowls to retrieve a reward. In this stage of the task, both bowls were rewarded and animals were allowed to retrieve the reward from both bowls before access was terminated. Animal went through a number of trials with the reward being hidden deeper in the sand across trials. In initial trials there was no sand in the bowls, in intermediate trials the pellets were placed on the top of the unscented sand, and in the final trials the reward pellets were placed at the bottom of the bowls (~1 cm below the surface of the unscented sand). This training phase was considered complete once the animals had successfully retrieved both rewards from below the sand over two consecutive trials. This habituation and training phase took the animals’ 8-12 trials to complete.

*1.2 Odor Habituation and Novel Odor Discrimination*

On the second day of the task (training day 2), animals were habituated to the different odors and then learned a novel association between scent and the sucrose pellet reward (Discrimination 1). During odor habituation the animals were presented with two bowls filled with scented sand, one bowl from each of the pre-defined odor pairs (supplemental material, Table S1), and allowed to retrieve the reward from both bowls. To prevent any potential for location (side of presentation) bias, the side of the initial odor presentation was randomised across animals for each odor pair and the odor pairs were presented across two trials in the different locations at each trial (e.g., left side presentation on trial 1, right side presentation on trial 2). Thus, animals encountered each odor pair twice, and completed odor habituation over six trials in total. The order of odor pair presentation was also randomised.

After odor habituation had been completed, animals went on to the novel odor discrimination phase (Discrimination 1). The animal was presented with one odor pair during this phase of the task, with only one of the odors rewarded. The odor pair allocation for each animal was randomised, as was the odor being rewarded and the initial location of the rewarded bowl. From then on the location of reward bowl presentation was pseudo-randomised, with a limit of two consecutive presentations at the same location. In the first four trials animals were allowed to dig in both bowls, and trials were not terminated until animals had dug in each bowl. The trial was terminated by preventing access to the bowls by closing the doors to the “choice” area once animals have moved into the holding area. From the fifth trial onwards, animals were only allowed to dig in one bowls, with access to the other bowl prevented once the animal had committed a dig. A dig was defined as breaking the surface of the sand with two consecutive movements of the forepaws. Therefore, from the fifth trial onwards, if the animal made an incorrect choice by initiating a dig in the unrewarded bowl, the animal received a time out and was not able to retrieve the reward for that trial. When an incorrect choice was made no limit was placed on how long the mouse was allowed to dig, to ensure that mice became aware that the bowl did not contain a reward. Access to the bowl was only limited once animals had moved into the holding area. During this phase of the task the latency to dig from the start of the trial (seconds), and whether the first dig choice was correct or incorrect were recorded. The animal was judged to have learned the odor-reward association when it had reached the criteria of making six consecutive correct choices across trials. A maximum limit of 40 trials was applied to this phase of the task, with animals reaching criteria on average in 17 trials. There is a 1.56% chance of animals performing six consecutive correct choice trials by random choice selection. This criterion is aligned with that previously applied in similar studies (Birrell and Brown, 2000; Dawson et al., 2012; Young et al., 2010; Tanaka et al., 2011).

*1.3 Second Odor Discrimination and Reversal Learning*

On the third day of the OB-DaRL test, the animals were presented with a new odor pair and the animal learned the second odor-reward discrimination (Discrimination 2) following the same protocol applied during Discrimination 1. Again, the latency to initiate a dig (seconds) and whether a dig was a correct or incorrect choice was recorded. Once the animal had made six consecutive correct choices, set as the criterion indicating that the odor-reward association had been learned, the animal then moved immediately to the next phase of the task, the reversal-learning phase.

During the reversal-learning phase the previously unrewarded odor within the odor pair was now rewarded, and vice versa. During this phase of the test the first four trials were again “free choice” trials, with the animal allowed to dig in both bowls in order to retrieve the reward from the newly rewarded odor. From trial five onwards animals were limited to digging in only one bowl, with the trial unrewarded if the animal initiated a dig in the unrewarded odor. During these trials the latency (seconds) to make a dig and whether the dig was correct or incorrect was recorded. The criteria for learning the new odor-reward association was set at six consecutive correct trials. A maximum limit of 60 trials was applied to reach criterion in this phase of the task.

At each phase of the task, correct and incorrect choices and the latency to make a dig were recorded. In addition, the correction ratio, the proportion of trials that were correct choices made after a previously punished (exclusion from reward collection and a time out) incorrect choice, was also calculated. The correction ratio was only recorded from the fifth trial onwards. Group sizes for the OB-DaRL task were WT *n*=29 (male, *n*=16) and *Nrxn1α^+^*^/−^ *n*=26 (male, *n*=13).

1. **^14^C-2-DG functional brain imaging**

14C-2-DG functional brain imaging was conducted in accordance with published protocols (Hughes et al., 2020; Openshaw et al., 2020). In brief, constitutive local cerebral glucose utilization (LCGU) measurement was initiated by injection of 14C-2-DG (intraperitoneal [i.p.]), 4.625 MBq/kg in physiological saline at 2.5 mL/kg (American Radiolabelled Chemicals Inc., USA). Animals are unanaesthetised and freely-moving during this protocol, and are returned to the home cage following this injection. At 45 min after 14C-2-DG injection animals were decapitated and a terminal blood sample collected, by torso inversion, into heparinized weigh boats. Plasma glucose concentrations (mmol/L) were detected from whole blood (Accu-Chek Aviva glucose monitor). The brain was then rapidly dissected out intact, frozen in isopentane (−40°C), and stored at (−80°C) until sectioning. Blood samples were centrifuged to isolate plasma, and plasma 14C concentrations (20 μL) were determined by liquid scintillation analysis (Packard). Frozen brains were sectioned (20 μm) in the coronal plane in a cryostat (−20°C). A series of three consecutive sections were retained from every 60 μm, thaw mounted onto slide covers, and rapidly dried on a hotplate (70°C). Autoradiograms were generated by apposing these sections, together with pre-calibrated 14C standards (39–1098 nCi/g tissue equivalents, American Radiolabelled Chemicals Inc., USA) to X-ray film (Carestream BioMax MR film, Sigma-Aldrich, UK) for 7 days. Autoradiographic images were analyzed by a computer-based image analysis system (MCID/M5+, Interfocus). The local isotope concentration for each brain region of interest (RoI) was derived from the optical density of the autoradiographic images relative to that of the co-exposed 14C standards. The experimenter undertaking these measurements is blind to the genotype and sex of the samples that they analyse. LCGU was determined in 39 brain regions of interest (RoI) across a range of neural systems (supplemental material, Table S2) with reference to a stereotaxic mouse brain atlas (Paxinos and Franklin, 2001). Within in each animal, 8-12 technical replicate measurements are taken for each RoI across 4-6 sections, and the average ^14^C concentration determined. The rate of LCGU, in each RoI, was determined as the ratio of 14C present in that region relative to that of the whole brain 14C concentration in the same animal, referred to as the 14C-2-DG uptake ratio. Group sizes were WT: n = 19 (male n = 9) and Nrxn1α+/−: n = 20 (male n = 10).

1. **Insulin Receptor Signalling Characterisation Using Western Blotting**

We used Western blotting to characterize insulin receptor signalling in the PFC and hippocampus of *Nrxn1α*^+/-^ mice (WT, n=6 [male, n=3]; *Nrxn1α*^+/-^ n=8 [male, n=4]). Total protein extracts from PFC and hippocampal tissue were prepared in radioimmunoprecipitation assay (RIPA) buffer (pH 7.4, 20ul/mg tissue) supplemented with phosphatase (10ul/ml, Sigma-Aldrich, Cat. No. P5726) and protease (5ul/ml, Sigma-Aldrich, Cat. No. P8340) inhibitors, centrifuged (10,000g at 4^o^C for 10 mins) and the supernatant removed. Protein levels were determined by Bradford assay. 20ug of protein, in laemmli buffer containing 5% β-mercaptoethanol, was denatured at 95^o^C for 5 minutes. Protein samples were resolved on Any kD™ Mini-PROTEAN® TGX™ Precast Protein stain free gels (Bio-Rad Laboratories Ltd, Cat. No. 4568126) at 180V for 40 minutes. Immediately following electrophoresis, the gel was UV-activated using the ChemiDoc MP imaging System (Bio-Rad Laboratories Ltd, Cat. No. 17001402). Following electrophoresis and gel imaging, the proteins were transferred via the TransBlot Turbo semi-dry blotting apparatus (Bio-Rad Laboratories, Cat. No. 1704150) onto nitrocellulose membranes (Bio-Rad Laboratories Ltd, No. 162–0115) and a total protein load image was captured using Image LabSoftware (Bio-Rad Laboratories Ltd). Membranes were blocked with 2% bovine serum albumin (BSA) in 1x TBST (TBS + 0.05% Tween) buffer at RT for 30 minutes, and probed with primary antibody overnight at 4^o^C. The primary antibodies used were for the insulin receptor (beta subunit: IRβ, Merck Millipore, Cat. No. 07-724, 1:1000) and the phosphorylated/activated IR (tyrosine 972 phosphorylated, IR pTyr972/pIR, Merck Millipore, Cat. No. 07-838, 1:1000). Following primary antibody incubation, blots were incubated with the HRP-linked secondary antibody (Goat Anti-rabbit IgG, HRP-linked Antibody Cat. No. 7074S, Cell Signalling, 1:2000) for 1 hour at RT. Membranes were developed with ECL Prime western blotting detection reagent kit (Amersham) and then images acquired with Chemi-Doc MP system and analyzed using Image Lab software (Bio-Rad Laboratories Ltd) that permits the normalization of a specific protein signal with total protein load. Data were then normalized to same-sex wild-type control.

**References**

Birrell J.M., Brown V.J. (2000). Medial frontal cortex mediates perceptual attentional set shifting in the rat. *J Neurosci*, 20, 4320-4. <https://doi.org/10.1523/jneurosci.20-11-04320.2000>.

Dawson N., Thompson R.J., McVie A., Thomson D.M., Morris B.J., Pratt J.A. (2012). Modafinil reverses phencyclidine-induced deficits in cognitive flexibility, cerebral metabolism, and functional brain connectivity. *Schizophr Bull*, 38, 457-74. <https://doi.org/10.1093/schbul/sbq090>.

Hughes R.B., Whittingham-Dowd J., Simmons R.E., Clapcote S.J., Broughton S.J., Dawson N. (2020). Ketamine restores thalamic-prefrontal cortex functional connectivity in a mouse model of neurodevelopmental disorder-associated 2p16.3 deletion. *Cereb Cortex,* 30, 2358-71. <https://doi.org/10.1093/cercor/bhz244>.

Openshaw R.L., Thomson D.M., Thompson R., Penninger J.M., Pratt J.A., Morris B.J., *et al.* (2020). Map2k7 haploinsufficiency induces brain imaging endophenotypes and behavioral phenotypes relevant to schizophrenia. *Schizophr Bull*, 46, 211-23. <https://doi.org/10.1093/schbul/sbz044>.

Paxinos G, Franklin KBJ. 2001. The mouse brain in stereotaxic coordinates. 2nd ed. London, UK: Academic Press.

Tanaka S., Young J.W., Gresack J.E., Geyer M.A., Risbrough V.B. (2011). Factor analysis of attentional set-shifting performance in young and aged mice. *Behav Brain Funct,* 7, 33. <https://doi.org/10.1186/1744-9081-7-33>.

Young J.W., Powell S.B., Geyer M.A., Jeste D.V., Risbrough V.B. (2010). The mouse attentional-set-shifting task: a method for assaying successful cognitive aging? *Cogn Affect Behav Neurosci,* 10, 243-51. <https://doi.org/10.3758/cabn.10.2.243>
